# Metabolomic Insights on Obesity and Diabetes from Feeding Diets Varying in Carbohydrate–Fat Ratios in Zucker Diabetic Fatty (ZDF) and Lean Zucker (Z_lean_) Rats

**DOI:** 10.3390/ijms27157017

**Published:** 2026-08-04

**Authors:** Mohd Naeem Mohd Nawi, Ranina Radzi, Azizan Ali, Siti Zubaidah Che Lem, Azlina Zulkapli, Ezarul Faradianna Lokman, Mansor Fazliana, Fatin Saparuddin, Norazlan Mohmad Misnan, Sreelakshmi Sankara Narayanan, Karuthan Chinna, Mohd Fairulnizal Md Noh, Zulfitri Azuan Mat Daud, Tilakavati Karupaiah

**Affiliations:** 1 Nutrition, Metabolism and Cardiovascular Research Centre, Institute for Medical Research, National Institutes of Health, Ministry of Health, Setia Alam 40170, Malaysia; naeem@moh.gov.my (M.N.M.N.);; 2 Good Laboratory Practice Section, Herbal Medicine Research Centre, Institute for Medical Research, National Institutes of Health, Ministry of Health, Kuala Lumpur 50588, Malaysia; 3 Laboratory Animal Resource Unit, Special Resource Centre, Institute for Medical Research, National Institutes of Health, Ministry of Health, Kuala Lumpur 50588, Malaysia; 4 Manager’s Office, National Institutes of Health, Ministry of Health, Setia Alam 40170, Malaysia; 5 Herbal Medicine Research Centre, Institute for Medical Research, National Institutes of Health, Ministry of Health, Setia Alam 40170, Malaysia; 6 School of Biosciences, Faculty of Health and Medical Sciences, Taylor’s University, Subang Jaya 47500, Malaysia; 7 Centre for Active Living, Taylor’s University, Subang Jaya 47500, Malaysia; 8 Institute of Research, Development and Innovation, IMU University, Kuala Lumpur 57000, Malaysia; 9 Department of Dietetics, Faculty of Medicine and Health Sciences, Universiti Putra Malaysia, Serdang 43400, Malaysia; 10 Jeffrey Cheah School of Medicine & Health Sciences, Monash University Malaysia, Jalan Lagoon Selatan, Bandar Sunway 47500, Malaysia

**Keywords:** metabolomics, obesity, diabetes, dietary, metformin

## Abstract

In population health the highly cited Atherosclerosis Risk in Communities study indicated a U-shaped association between carbohydrate intake and mortality, whilst the Prospective Urban Rural Epidemiology study linked higher fat intake to lower mortality risks. The Malaysia Lipid Study reported high-fat and high-carbohydrate dietary patterns were associated with increased cardiometabolic risks, including insulin resistance and small dense LDL particles generation. This animal model study therefore was purposely designed to evaluate metabolic outcomes of carbohydrate–fat permutations in Zucker diabetic fatty (ZDF) and Zucker lean (Z_lean_) rats by using ^1^H Nuclear Magnetic Resonance (NMR) metabolomics. Twenty-four ZDF rats were randomly divided into four groups (*n* = 6 per group): control (standard diet), Diet A (54%-energy carbohydrate, 32%-energy fat, 14%-energy protein) mimicking a recommended adult Malaysian diet, Diet B (49%-energy carbohydrate, 37%-energy fat, 14%-energy protein) mimicking a low-carbohydrate, moderate high-fat diet, and metformin treatment (100 mg/kg), which effectively represents a 5%-energy exchange in isocaloric meals. An additional six Z_lean_ were given Diet A (*n* = 3) and Diet B (*n* = 3). The intervention lasted eight weeks. Using log-transformed data, analysis of variance (ANOVA) revealed significant differences between the groups for eleven metabolites (1,6-anhydro-β-D-glucose, 2-hydroxyvalerate, acetate, 3-aminoisobutyrate, 3-hydroxybutyrate, carnitine, choline, citrate, creatine, lactate, and N-methylhydantoin) (all *p* < 0.05) which remained significant even after false discovery rate (FDR) correction. Majorly elevated metabolites in both ZDF and Z_lean_ rats were 3-hydroxybutyrate, N-methylhydantoin, 3-aminoisobutyrate, and carnitine, indicating dietary influences independent of diabetes status. Conversely, citrate and 1,6-anhydro-β-D-glucose levels showed distinct patterns across the groups, with Z_lean_ rats exhibiting lower levels and ZDF rats showing higher levels compared to healthy controls, suggesting potential genetic or physiological influences. Other metabolites such as creatine, acetate, choline and 2-hydroxyvalerate showed varied trends, highlighting metabolic complexities. Compared to the control group, metformin treatment generally resulted in lower levels of metabolites, except for acetate, which was higher, indicating improved insulin sensitivity. The findings indicated moderate high-fat diets may exacerbate metabolic disturbances as seen in both ZDF and Z_lean_ rats, while metformin treatment generally improved metabolic profiles.

## 1. Introduction

In 2025, Malaysia carried the highest diabetes prevalence rate of 19.9% in Southeast Asia [1] whilst earlier data from the National Health and Morbidity Survey (NHMS) 2023 indicated that the increasing trend for high blood glucose [2,3] is a major public health concern. Alongside, the key non-communicable disease (NCD) risk indicators flagged for Malaysia, namely high blood pressure, high blood glucose, dietary risks, and high body mass index (BMI) [4], were underpinning 63% of its NCD-linked mortality whilst contributing to 65% of Southeast Asia’s burden [5]. White rice and sugar were the two most consumed foods daily by Malaysian adults as indicated by the Malaysian Adult Nutrition Survey (MANS) in 2014 [6]. The more recent NHMS 2024 indicated 47.0% of Malaysian adults exceeded the World Health Organization (WHO) recommendations of >7.5 teaspoons/day [7]. Half of total sugar intake originated from sugar-sweetened beverages (SSBs) with 59% of adults reportedly drinking more than one serving of SSBs daily [7]. Given that white rice, a refined grain with a high glycaemic index, and excessive sugar intake are linked to obesity, type 2 diabetes mellitus (T2DM), and cardiovascular disease (CVD), these consumption patterns highlight poor dietary adherence and the growing dietary risks contributing to the national burden of NCDs [8,9,10].

Large cohort studies such as Atherosclerosis Risk in Communities (ARIC) [11] and Prospective Urban Rural Epidemiology (PURE) [12] are suggesting both very low and very high carbohydrate intake potentially increase cardiovascular mortality, whereas moderate carbohydrate and relatively higher fat intakes are associated with lower risk [11]. In Malaysia, the Malaysia Lipid Study linked high-fat, high-carbohydrate diets to insulin resistance, atherogenic lipoprotein patterns and dyslipidaemia [13]. Although protein supports weight and body composition, its direct metabolic effects appear modest compared with those of carbohydrate and fat and may act mainly via indirect improvements in insulin sensitivity [14] and cardiovascular markers [15]. In this context, clarifying how varying dietary carbohydrate and fat intakes shape metabolic health is critical.

Dietary macronutrient composition consistently influences circulating metabolite patterns in both human and animal studies. Esko et al. [16] showed that metabolomic profiles could differentiate between controlled high-fat, high-carbohydrate and other macronutrient-specific diets, allowing objective assessment of dietary composition and adherence in clinical nutrition trials. Wallenius et al. [17] observed that, in humans, an isoenergetic high-fat diet produced a plasma metabolite profile enriched in branched-chain amino acids and related metabolites, indicating a shift toward reduced insulin sensitivity even when short-term glycaemic and insulinemic responses are comparable to those seen with a high-carbohydrate diet. In rodent models, Dankel et al. [18] reported that high-fat feeding preferentially increased hepatic lipid content and specific lipid-associated metabolites, whereas high-carbohydrate feeding more strongly augmented hepatic glucose and lipogenesis-related metabolites together with changes in key lipogenic enzymes. Systematically altering dietary carbohydrate and fat ratios in male Wistar rats generated distinct lipidomic and metabolomic signatures, with carbohydrate-rich diets promoting de novo lipogenesis and higher plasma triglycerides, and fat-rich diets favouring hepatic fatty acid accumulation without parallel rises in circulating triglycerides [19].

Advancements in nutritional metabolomics, particularly through the use of ^1^H nuclear magnetic resonance (NMR) spectroscopy, provides an advanced approach to characterising the molecular phenotypes of individuals. This technique enables a detailed analysis of how individuals respond to different dietary compositions and offers a comprehensive assessment of their mechanistic and predictive roles in health. One study identified 152 metabolites with concentrations that varied significantly by diet [16], while another found that adherence to healthy dietary patterns correlated with specific metabolite profiles associated with the risk of prediabetes and T2DM [20]. Additionally, a separate study demonstrated significant differences in metabolite concentrations that correlated with dietary composition [21]. In line with these findings, recent ^1^H NMR metabolomics studies by Saparuddin et al. [22] and Fazliana et al. [23] have shown that serum and plasma metabolite profiles can distinguish metabolically healthy from unhealthy obese and diabetic individuals, demonstrating that NMR-based metabolomics can stratify diet-related metabolic risk beyond traditional measures such as BMI.

These observations suggest that defined changes in dietary macronutrient composition should produce characteristic alterations in circulating metabolites and metabolic pathways, particularly those related to amino acid metabolism, ketone body production, lipid handling and glucose regulation. The use of metabolomics presents a compelling opportunity to investigate dietary patterns with greater precision and detail. These dietary patterns can significantly influence metabolic health, either promoting improvement or contributing to dysregulation, which is particularly relevant in populations at risk for T2DM. The Zucker Diabetic Fatty (ZDF) rat model, known for its genetic predisposition to obesity and T2DM, serves as an invaluable tool for examining the metabolic consequences of dietary interventions. ZDF rats carry a specific genetic mutation (*fa*/*fa*) that leads to obesity and insulin resistance, offering a stable and controlled environment to explore the pathophysiology of these conditions [24,25,26]. This genetic predisposition enables researchers to investigate the underlying mechanisms of obesity and diabetes without the confounding variables present in human populations, such as lifestyle and environmental factors [27]. Building on prior human and rodent metabolomics studies that have identified distinct signatures for high-carbohydrate and high-fat diets, the present study was designed to test the hypothesis that isocaloric manipulation of the dietary carbohydrate-to-fat ratio in ZDF rats would yield differential plasma metabolite profiles and pathway perturbations. Specifically, that higher-carbohydrate, lower-fat meals would favour elevations in glucose, lactate and lipogenesis-related metabolites, whereas higher-fat, lower-carbohydrate meals would favour increased ketone bodies and altered fatty acid-derived metabolites. This study thus aimed to investigate the effects of carbohydrate–fat permutations on mediating metabolic outcomes by administering isocaloric meals to Zucker diabetic fatty (ZDF) rats using ^1^H NMR metabolomics.

## 2. Results

**Figure 1:**
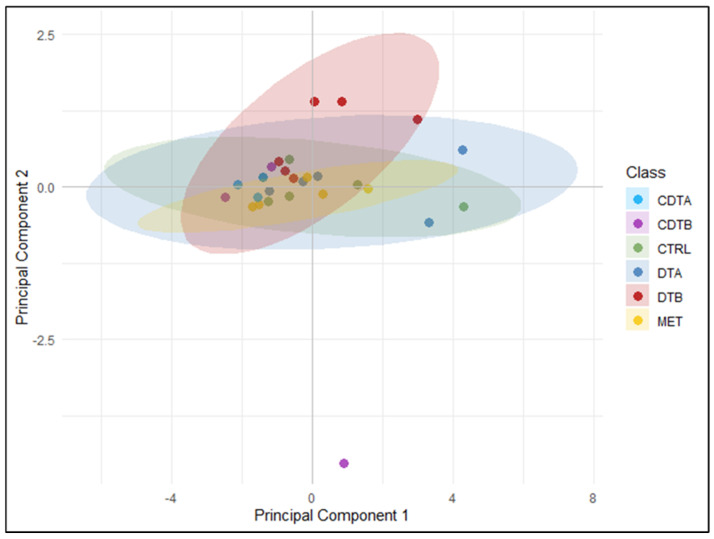
PCA score plot for all groups (Light blue-Z_lea_n rats with Diet A, CDTA; purple-Z_lean_ rats with Diet B, CDTB; green-ZDF control, CTRL; blue-ZDF rats with Diet A, DTA; red-ZDF rats with Diet B, DTB; yellow-ZDF rats treated with metformin, MET).

The principal component analysis (PCA) score plot across all six groups (Figure 1) revealed that the model accounted for 65.6% of the variation and 40.5% of the predictive variation, indicating a good model fit (*R*^2^ = 0.656) and acceptable predictive performance (*Q*^2^ = 0.405). Diet A was formulated to represent a mixed ‘normal human’ pattern (54% carbohydrate, 32% fat, 14% protein), and Diet B a relatively low-carbohydrate, moderate high-fat pattern (49% carbohydrate, 37% fat, 14% protein).

Z_lean_ rats receiving Diet A (light blue) and Diet B (purple) clustered mainly near the centre of the score plot, with one Z_lean_ Diet B sample appearing as an outlier on principal component 1. ZDF control rats (green) overlapped partially with Z_lean_ rats but tended to shift toward positive scores on principal component 1, while ZDF rats fed Diet A (blue) and Diet B (red) showed a more pronounced displacement, indicating diet-related metabolic differences within the diabetic phenotype. ZDF rats treated with metformin (yellow) occupied an intermediate region, overlapping both control and diet-treated ZDF groups, suggesting that metformin partially normalised the metabolic profile toward that of the other groups. Ellipses represented the 95% confidence regions for each group to aid visualisation of group-level clustering.

**Figure 2:**
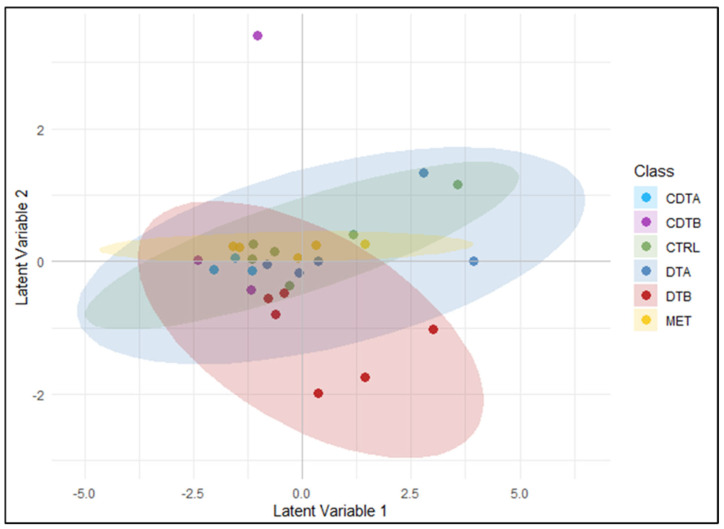
PLS-DA score plot for all groups (Light blue-Z_lean_ rats with Diet A, CDTA; purple-Z_lean_ rats with Diet B, CDTB; green-ZDF control, CTRL; blue-ZDF rats with Diet A, DTA; red-ZDF rats with Diet B, DTB; yellow-ZDF rats treated with metformin, MET).

Partial least squares-discriminant analysis (PLS-DA) for all six groups revealed group-specific tendencies as reflected by group centroids and confidence intervals (Figure 2). For this model, the cumulative explained variance was *R*^2^*X* = 0.641 and *R*^2^*Y* = 0.154, with a cross-validated predictive ability of *Q*^2^ = −0.022, indicating that although the latent variables captured a substantial proportion of the variance in the metabolite data, they accounted for only a modest fraction of the variance in group labels and conferred negligible predictive power. Consistent with this, analysis of variance of cross-validated residuals (CV-ANOVA) yielded a non-significant *p*-value of 1.0, indicating that the supervised model did not perform better than a null model. Because sample sizes were limited and class sizes were imbalanced, permutation testing was not applied to the full six-group model; instead, permutation analyses restricted to control-related contrasts yielded low *R*^2^ and negative *Q*^2^ intercepts (*R*^2^ = 0.158, *Q*^2^ = −0.060), supporting the absence of pronounced overfitting and reinforcing that the observed separation should be regarded as descriptive. Overall, these diagnostics indicated that, although the PLS-DA score plot suggested weak group-specific structure, the supervised model offered only limited, non-robust discrimination and was therefore used solely for exploratory visualisation rather than for definitive biomarker selection.

With multivariate PLS-DA analysis, Diet B (red) demonstrated the most distinctive metabolic profile, forming a separate cluster with minimal overlap with the other treatment groups. Control samples (green) showed high intra-group consistency while Diet A (blue) and metformin (yellow) treatments displayed more diffuse clustering patterns with substantial overlap. Notably, lean Zucker (Z_lean_) rats administered either Diet A or Diet B clustered predominantly with the latter groups, barring one outlier rat.

**Figure 3:**
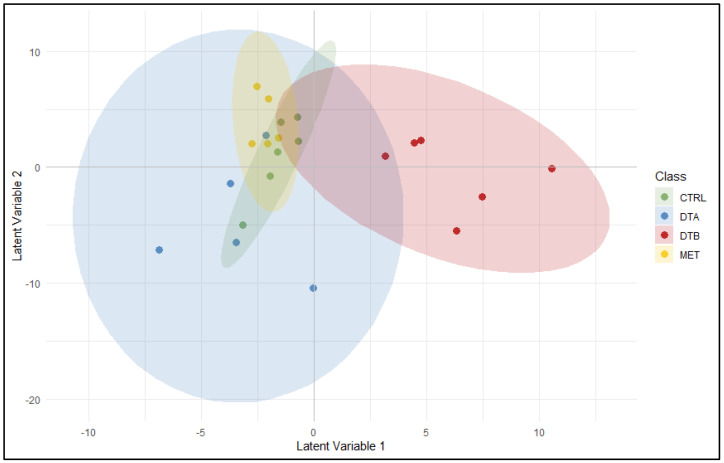
PLS-DA score plot for ZDF rats (green-ZDF control, CTRL; blue-ZDF rats with Diet A, DTA; red-ZDF rats with Diet B, DTB; yellow-ZDF rats treated with metformin, MET).

PLS-DA was conducted in ZDF rats only (control, Diet A, Diet B, and metformin), and the corresponding score plot is shown in Figure 3. The cumulative explained variance was *R*^2^*X* = 0.650 and *R*^2^*Y* = 0.292, indicating that the latent variables captured considerable variation in the metabolite data but explained only a modest proportion of the variation in treatment labels. The cross-validated predictive ability was poor (*Q*^2^ = −0.131), and CV-ANOVA yielded a non-significant *p*-value of 1.0, indicating no improvement over a null model. Permutation diagnostics produced low *R*^2^ and slightly negative *Q*^2^ intercepts (*R*^2^ = 0.272, *Q*^2^ = −0.014), suggesting limited risk of overfitting but also that the apparent separation could readily occur under random class assignment. In line with the six-group analysis, this model was therefore interpreted as descriptive: the score plot was used to illustrate multivariate tendencies, whereas formal classification and biomarker selection relied on univariate statistics rather than on PLS-DA outputs.

Based on this PLS-DA score plot, Diet B exhibited the most distinctive metabolic profile, forming a clearly separated cluster along latent variable 1 with minimal overlap with the other treatment groups, indicating a pronounced shift in the underlying metabolome relative to the remaining interventions. Control ZDF rats (green) form a compact cluster with tight 95% confidence ellipses, consistent with high intra-group homogeneity, whereas Diet A–treated ZDF rats (blue) and metformin-treated ZDF rats (yellow) occupy overlapping regions of the latent space and show more diffuse clustering, suggesting more heterogeneous metabolic responses and substantial similarity between these two treatments.

**Figure 4:**
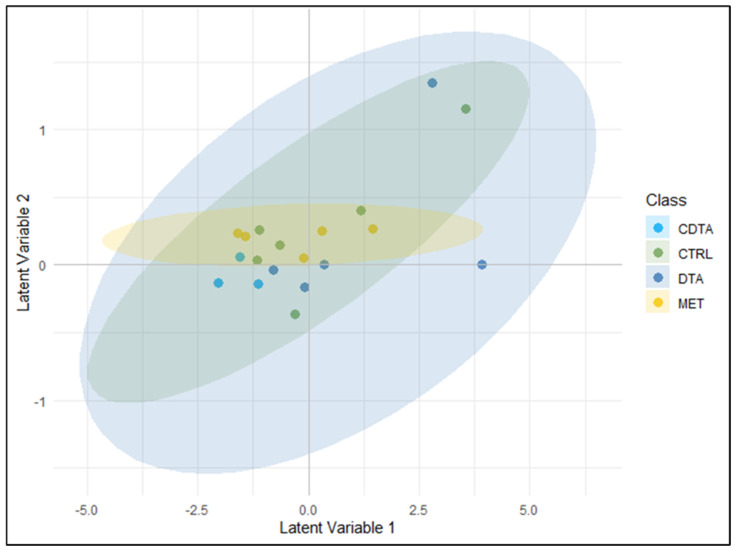
PLS-DA score plot for Diet A (light blue-Z_lean_ rats with Diet A, CDTA; green-ZDF control, CTRL; blue-ZDF rats with Diet A, DTA; yellow-ZDF rats treated with metformin, MET).

For the PLS-DA model restricted to four treatment groups (control, Diet A, metformin, and Z_lean_ Diet A), the cumulative explained variance was *R*^2^*X* = 0.334 and *R*^2^*Y* = 0.405, indicating that the model detected some structure in the metabolite data but still offered only a modest fit to the treatment labels. The cross-validated predictive ability was poor (*Q*^2^ = −0.089), CV-ANOVA was non-significant (*p* = 1.0), and permutation testing centred on control-related contrasts yielded regression intercepts of 0.541 for *R*^2^ and −0.038 for *Q*^2^, consistent with negligible predictive performance under random class assignment. In view of these metrics, this PLS-DA configuration was likewise used chiefly to illustrate treatment-related trends in multivariate space rather than as a basis for inferential classification or biomarker discovery. The corresponding score plot, with 95% confidence ellipses, depicts the group-level dispersion and overlap between control, Diet A, metformin, and Z_lean_ Diet A (Figure 4).

The plot showed considerable overlap between groups, particularly between Diet A (blue) and metformin (yellow) which displayed diffuse clustering patterns in the central region along latent variable 1. The control group (green) demonstrated relatively consistent positioning, primarily distributed in the upper-right quadrant with positive values on both latent variables, though showing some variability. The Z_lean_ Diet A group (light blue) clustered mostly in the central to left region of the plot. The confidence ellipses indicated considerable overlap in metabolic profiles between the four displayed groups, suggesting similarities in their metabolic responses despite different treatments.

**Figure 5:**
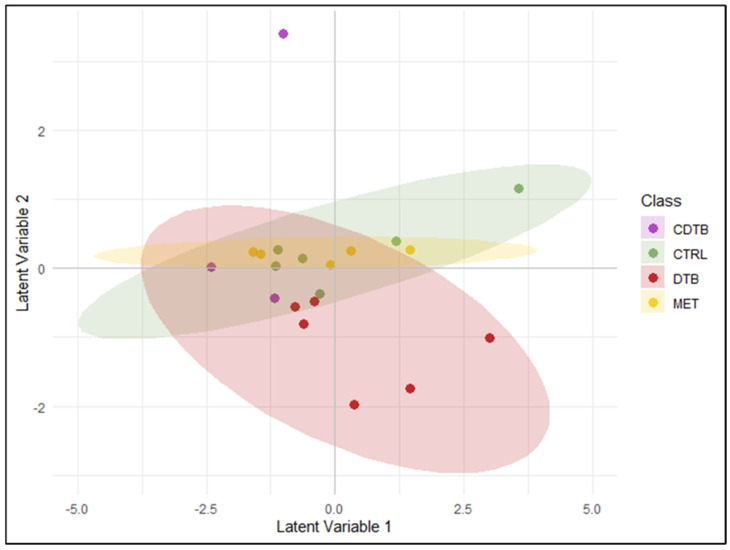
PLS-DA score plot for Diet B (purple-Z_lean_ rats with Diet B, CDTB; green-ZDF control, CTRL; red-ZDF rats with Diet B, DTB; yellow-ZDF rats treated with metformin, MET).

A supervised PLS-DA model was constructed to examine multivariate differences among control, Diet B, metformin, and Z_lean_ Diet B. The model captured a moderate proportion of variance in the metabolite data (*R*^2^*X* = 0.529) but only modestly explained variation in treatment labels (*R*^2^*Y* = 0.314). Cross-validated predictive ability remained poor (*Q*^2^ = 0.06), CV-ANOVA was non-significant (*p* = 0.831), and permutation testing involving the control group yielded intercepts of 0.212 for *R*^2^ and −0.086 for *Q*^2^. Collectively, these findings indicated that, although multivariate structure was present, the model lacked robust predictive capacity and was therefore used as a descriptive tool to visualise treatment-related clustering rather than for formal classification or biomarker discovery. The corresponding score plot, together with 95% confidence ellipses, illustrates the relative separation and overlap between the four groups (Figure 5).

In this analysis, the Diet B group (red) exhibited the most distinctive metabolic profile, forming a separate cluster primarily in the lower right quadrant of the plot with minimal overlap with other groups. The control group (green) demonstrated relatively high intra-group consistency, with samples clustering together in the upper-right region. The metformin group (yellow) displayed a more diffuse clustering pattern, with its confidence ellipse overlapping with both the control and Diet B, indicating some metabolic similarity or transitional profiles. The Z_lean_ Diet B group (purple) was represented by a small number of samples, with one notable outlier positioned distant off the main cluster, suggesting greater variability or a unique metabolic response within this group.

The evaluation of biomarkers was performed using orthogonal partial least squares-discriminant analysis (OPLS-DA) score plots, variable importance in projection (VIP) scores, loadings plots, and S-plots, with results summarised in the Supplementary Materials. These OPLS-DA-derived metrics were used solely to visualise multivariate patterns and highlight candidate spectral features and were not themselves used as definitive criteria for statistical significance or biomarker selection. As per the Human Metabolome Database (HMDB), 44 metabolites were identified. In Table 1 mean concentrations of metabolites were compared between the ZDF control, ZDF Diet A, ZDF Diet B, ZDF metformin, Z_lean_ Diet A and Z_lean_ Diet B groups. Using log-transformed data, analysis of variance (ANOVA) revealed significant differences between the groups for eleven metabolites namely 1,6-anhydro-β-D-glucose, 2-hydroxyvalerate, acetate, 3-aminoisobutyrate, 3-hydroxybutyrate, carnitine, choline, citrate, creatine, lactate, and N-methylhydantoin (all *p* < 0.05). However, because Z_lean_ groups had very small numbers of animals (*n* = 3), the assumptions and power of parametric ANOVA and post hoc Tukey-HSD tests were only weakly met for those comparisons; accordingly, results involving the Z_lean_ interventions, were reported as exploratory and should be regarded as preliminary.

To account for false discovery rate (FDR), a Benjamini–Hochberg correction was performed to rank metabolites based on their *p*-values. Table 2 displays the metabolites ranked after applying the B-H procedure with an FDR threshold of 10%. The top-ranked metabolites were 3-hydroxybutyrate, N-methylhydantoin, 3-aminoisobutyrate, creatine, acetate, carnitine, citrate, choline, 1,6-anhydro-β-D-glucose, 2-hydroxyvalerate, and lactate. All metabolites listed in Table 2 remained statistically significant after Benjamini–Hochberg adjustment at an FDR threshold of 10%, indicating that the observed associations are robust to multiple testing correction.

**Figure 6:**
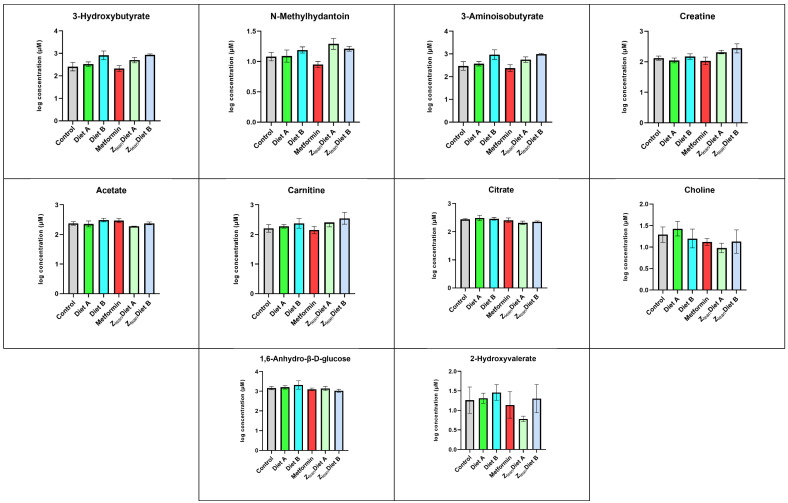
Top-ranked metabolite concentrations (grey-control; green-Diet A; cyan-Diet B; red-metformin; light green-Z_lean_ Diet A; light blue-Z_lean_ Diet B).

**Figure 7:**
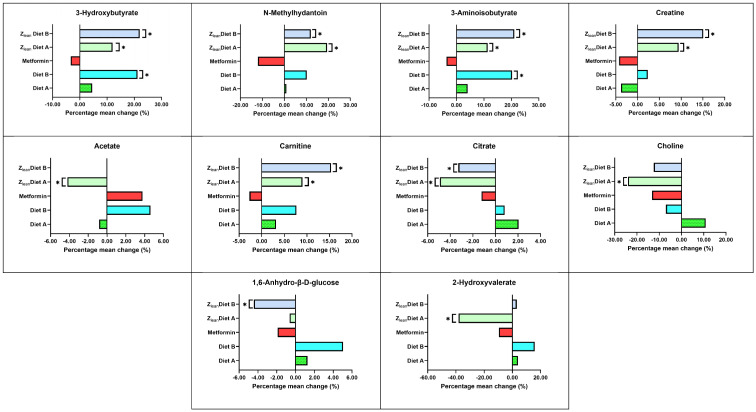
Percent change in top-ranked metabolites relative to control group (green-Diet A; cyan-Diet B; red-metformin; light green-Z_lean_ Diet A; light blue-Z_lean_ Diet B). Note-* Significance to control is at *p* < 0.05.

For clarity and readability, the significant metabolites are presented in two separate tables: [a] Table 3 lists the top ten metabolites for the ZDF groups, and [b] Table 4 lists those for the Z_lean_ groups compared to the control. However, the statistical analysis was conducted collectively across all groups to ensure a comprehensive comparison. Figure 6 shows the concentrations of these top-ranked metabolites, and Figure 7 depicts the percentage change in these metabolites compared to the control group.

For top-ranked metabolites (Table 5), distinct trends in metabolite concentrations were observed relative to the control group in terms of ZDF vs. Z_lean_ rats across treatments by Diet A, Diet B and metformin.

Compared to controls, ZDF rats on metformin experienced non-significantly decreased trends for all metabolite concentrations except for acetate. Comparatively, the following observations were related to ZDF or Z_lean_ rats on Diets A or B.

- Diets A and B induced in Z_lean_ rats significantly increased trends for 3-hydroxybutyrate, N-methylhydantoin, 3-aminoisobutyrate, creatine and carnitine whilst the 3-hydroxybutyrate and 3-aminoisobutyrate raising effects of Diet B were also significant for the ZDF rats on Diet B.
- Acetate reduction was mediated by Diet A treatment in both ZDF and Z_lean_ rats with significant reduction evident in the lean model; whereas an increasing non-significant trend was mediated for the ZDF rats on Diet B and metformin treatments.
- Significantly decreased citrate trends were mediated by Diets A and B in Z_lean_ rats and in ZDF rats on metformin treatment (not significant) as opposed to increased non-significant trends in ZDF rats consuming the diet treatments.
- Diet A mediated increased choline levels in ZDF rats compared to a significant reduction in the lean model. Diet B was associated with non-significant reductions in ZDF and Z_lean_ rats as with metformin-treated ZDF rats.
- Diets A and B mediated increased 1,6-anhydro-β-D-glucose in ZDF rats unlike metformin-treated ZDF rats and Z_lean_ rats which experienced reductions with Diet B producing a significant reduction in the lean model.
- Diet A mediated decreased 2-hydroxyvalerate levels in metformin-treated ZDF and Z_lean_ rats with the latter experiencing a greater significant reduction whereas non-significant increased trends were evident for the diabetes model on both diet treatments and the lean rat model on Diet B treatment.
- Because the Z_lean_ groups had very small sample sizes (*n* = 3), statistical results for these interventions are considered exploratory and should be interpreted as preliminary.

**Figure 8:**
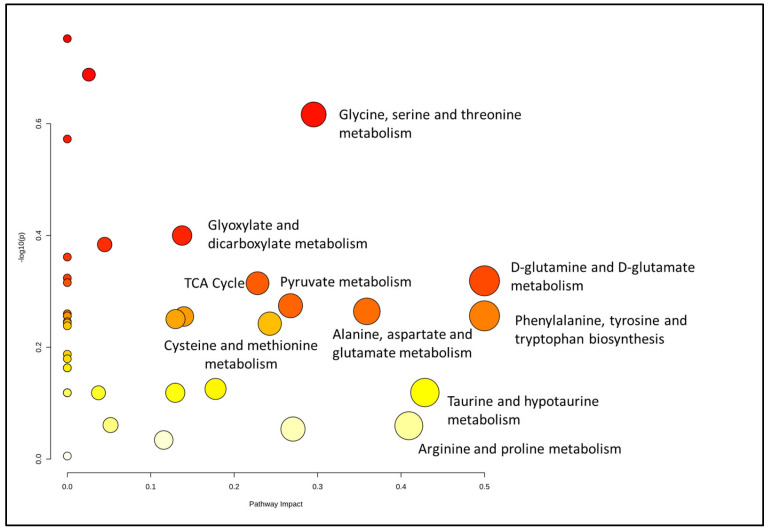
Pathway analysis Diet A to control. Circle size represents pathway impact, with larger circles indicating greater perturbation. Colour intensity ranges from yellow to red, where darker red denotes higher statistical significance (lower *p*-values).

**Figure 9:**
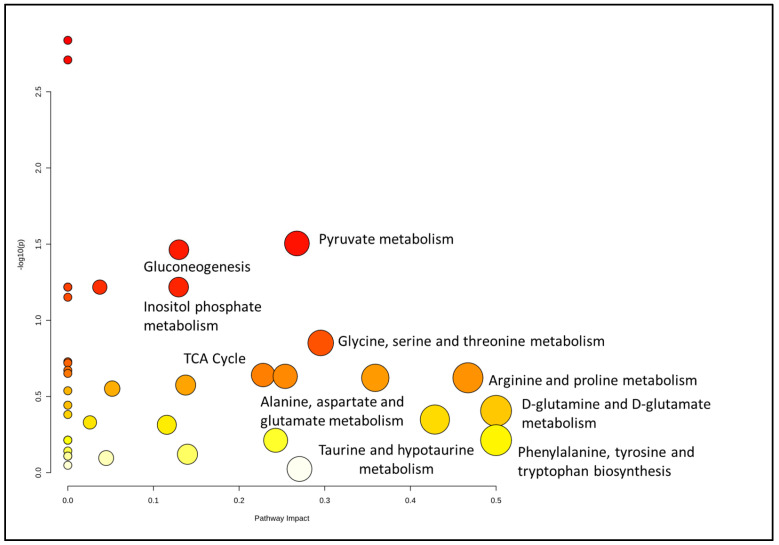
Pathway analysis Diet B to control. Circle size represents pathway impact, with larger circles indicating greater perturbation. Colour intensity ranges from yellow to red, where darker red denotes higher statistical significance (lower *p*-values).

**Figure 10:**
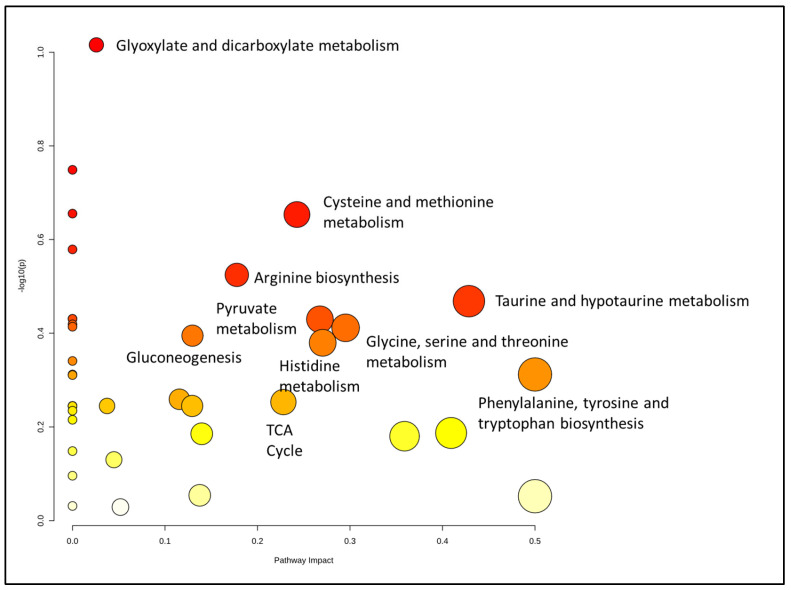
Pathway analysis metformin to control. Circle size represents pathway impact, with larger circles indicating greater perturbation. Colour intensity ranges from yellow to red, where darker red denotes higher statistical significance (lower *p*-values).

Figure 8, Figure 9 and Figure 10 depict the pathway analysis for ZDF rats receiving Diet A, Diet B, and metformin in comparison to the control group. The top three pathways differentiating Diet A from the control group were D-glutamine and D-glutamate metabolism (impact = 0.5, −log10(*p*) = 0.319), phenylalanine, tyrosine and tryptophan biosynthesis (impact = 0.5, −log10(*p*) = 0.256), and glycine, serine and threonine metabolism (impact = 0.295, −log10(*p*) = 0.616). In comparing Diet B to the control group, the pathways that emerged as distinguishing factors were pyruvate metabolism (impact = 0.268, −log10(*p*) = 0.617), glycine, serine, and threonine metabolism (impact = 0.295, −log10(*p*) = 0.852), and arginine and proline metabolism (impact = 0.467, −log10(*p*) = 0.623). Between metformin and the control group, the main pathways that were affected included phenylalanine, tyrosine, and tryptophan biosynthesis (impact = 0.5, −log10(*p*) = 0.312), cysteine and methionine metabolism (impact = 0.243, −log10(*p*) = 0.653), as well as taurine and hypotaurine metabolism (impact = 0.429, −log10(*p*) = 0.468).

## 3. Discussion

**Figure 11:**
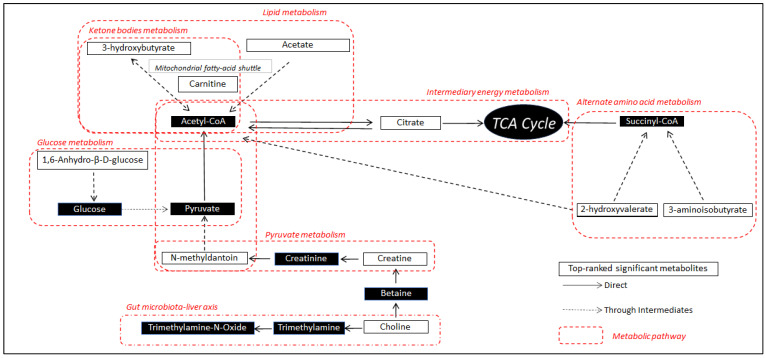
Pathway exploration of ZDF rats (in the metabolic pathway diagram, metabolites shown in white boxes represent the top-ranked significant metabolites identified in this study. Black boxes represent other components within the pathway. Solid arrows indicate direct metabolic pathways, while dashed arrows denote pathways that proceed through intermediaries).

This study examined the metabolic impacts of two dietary regimens which is Diet A, modelled after a normal human diet, and Diet B, a moderate high-fat diet on both ZDF and Z_lean_ rats. Diet A provided 32% of total energy from fat and 54% from carbohydrate, whereas Diet B increased fat to 37% and reduced carbohydrate to 49%, representing a deliberate, graded shift in macronutrient ratio rather than an extreme ketogenic challenge. Importantly, the pathway-level changes documented in this study emerged in response to a relatively modest isocaloric shift of approximately five percentage points in dietary fat and carbohydrate, suggesting that small changes in macronutrient distribution may be sufficient to remodel metabolic phenotypes in diabetic and lean models. This incremental increase in dietary fat and corresponding reduction in carbohydrate was chosen to probe whether relatively modest changes in energy substrate supply are sufficient to alter the metabolic phenotype in diabetic and lean models. Additionally, ZDF rats were studied under control conditions on a standard commercial chow and with metformin treatment as the standard pharmacological intervention for diabetes. Where possible, our interpretation draws on cited studies that employed more classical high-fat diet protocols and on phenotypic models of obesity or diabetes when such dietary regimens were not available. Multivariate analyses such as PLS-DA were performed to visualise overall clustering and separation between groups, while statistical inference was based on univariate tests. The present study’s findings reveal complex metabolic alterations across various pathways (Figure 11).

### 3.1. Treatment Effects on Ketogenic Pathway

The modest shift in carbohydrate-to-fat ratio between Diet A and Diet B is expected to alter hepatic substrate selection and mitochondrial energy handling, particularly in insulin-resistant ZDF rats. When carbohydrate availability is relatively lower and dietary fat intake higher, hepatic β-oxidation generates more acetyl-CoA, favouring ketone body formation (3-hydroxybutyrate, 2-hydroxyvalerate) and increasing reliance on alternative fuels when glucose utilisation is impaired [28]. In parallel, sustained lipid oxidation and increased anaplerotic input from amino acid catabolism can remodel mitochondrial tricarboxylic acid (TCA) cycle flux and redox status, while chronic exposure to higher fat loads tends to aggravate insulin resistance even as it stimulates ketogenesis [29]. Within this framework, the patterns observed for 3-hydroxybutyrate, 2-hydroxyvalerate and acetate in response to Diet A, Diet B and metformin can be interpreted as reflecting graded shifts in fuel preference and mitochondrial metabolism driven by macronutrient composition and pharmacological improvement of insulin sensitivity. The effects of Diet A, Diet B and metformin treatment in diabetic and lean rat models produced varying changes in 3-hydroxybutyrate and 2-hydroxyvalerate, metabolites involved in the ketogenic pathway and alternative energy production that are not exclusively dependent on diabetic pathophysiology. Alterations in ketone body and alternative energy metabolism reflect shifts in substrate utilisation under impaired glucose homeostasis. The metabolism of 3-hydroxybutyrate, a key ketone body, involves oxidation to acetoacetate and conversion to acetyl-CoA, which enters the TCA cycle [30]; this ATP-independent process [31] is often upregulated in diabetes due to impaired glucose absorption and insulin deficiency [32]. Elevated 3-hydroxybutyrate levels were observed with both dietary treatments, with the moderate high-fat Diet B likely contributing to more severe insulin resistance in both rat models, independent of diabetic status. Conversely, diabetic rats on metformin exhibited lower 3-hydroxybutyrate levels, albeit not statistically significant, consistent with metformin’s known effects on improving insulin sensitivity and enhancing glucose uptake, thereby reducing reliance on ketogenesis [33,34]. The metformin-associated changes in 3-hydroxybutyrate in the present study align with previous observations in high-fat-diet diabetic rats [35] but differ from findings in standard-diet diabetic rats, underscoring the complex interplay between diet, diabetes and metformin’s metabolic effects. Sajak et al. [36] also demonstrated variability in urinary 3-hydroxybutyrate among normal, obese and obese-diabetic rats, highlighting metabolic diversity across these phenotypes. While the present study analysed plasma 3-hydroxybutyrate, the cited studies focused on urinary levels, which may account for some discrepancies.

2-hydroxyvalerate is associated with branched-chain amino acid metabolism and impaired mitochondrial oxidation. As a C5 ketone body derived from odd-carbon fatty acids, it can serve as an alternative energy fuel for the brain and has been linked to lactic acidaemia, propionyl-CoA carboxylase deficiency [37] and type 1 diabetes in mice [38]. In the present study, elevated 2-hydroxyvalerate levels in ZDF rats receiving Diet A and Diet B may reflect increased ketone body production in response to poor glucose utilisation, as suggested by Lu et al. [39]. Conversely, lower 2-hydroxyvalerate levels in ZDF rats treated with metformin were indicative of improved glucose tolerance and insulin sensitivity, consistent with metformin’s known actions [40,41]. The contrasting response in Z_lean_ rats suggests diet-specific effects on 2-hydroxyvalerate metabolism, with Diet A, which has a more balanced macronutrient profile than the moderate high-fat Diet B, producing a significantly greater reduction in this metabolite, comparable to the metformin-associated decrease in ZDF rats. These observations are in line with reports identifying 2-hydroxyvalerate as a metabolite associated with obesity [42] and type 1 diabetes-related metabolic alterations [38]. Short-chain fatty acids, particularly acetate, are key mediators of host–microbiota interactions and energy metabolism. Acetate participates in energy generation, lipid synthesis and protein acetylation [43] and is converted to acetyl-CoA by acyl-CoA short-chain synthetases for cellular utilisation [44,45,46]. Its relationship with insulin sensitivity remains debated: studies have reported inverse associations with fasting insulin in obese women [47] and positive correlations with insulin sensitivity in morbidly obese individuals [48], whereas others have not confirmed these findings [49]. In the present study, ZDF rats on the moderate high-fat Diet B and on metformin exhibited higher acetate levels than controls, whereas ZDF rats on Diet A had lower levels. In Z_lean_ rats, acetate levels were significantly reduced with Diet A and unchanged with Diet B. Thus, Diet A was associated with decreased acetate in both models, while Diet B and metformin tended to increase acetate in ZDF rats, underscoring the complex regulation of acetate across metabolic states and interventions. The higher fat content of Diet B likely promoted a shift from lipogenesis toward fat oxidation in the context of diet-induced obesity [50,51], whereas elevated acetate in metformin-treated ZDF rats may reflect improved insulin sensitivity as reported elsewhere [47,48]. These findings partially align with previous work in diabetic and obese rat models: Sajak et al. [36] and Zhang et al. [52] reported increased acetate levels in diabetic rats, Guan et al. [53] observed elevated urinary acetate excretion in rats with prolonged diabetes and Zhao et al. [54] found higher acetate levels in obese Zucker rats compared with lean controls, while Goto-Kakizaki rats showed reduced levels relative to Wistar controls. Importantly, the present study analysed plasma acetate, whereas many prior studies focused on urinary concentrations, which may contribute to differences in the direction and magnitude of changes observed. Overall, the observation that metformin lowers 3-hydroxybutyrate while Diet B raises it is in keeping with improved insulin sensitivity versus diet-induced substrate shifts. Nonetheless, the findings of the present study are limited to plasma metabolites; tissue-level ketone and acetate fluxes were not measured, so the proposed mechanisms should be interpreted as biologically plausible but indirect.

### 3.2. Pyruvate Metabolism

Creatine metabolism plays a central role in cellular energy buffering and mitochondrial function, making it highly sensitive to metabolic disturbances. Changes in creatine and its downstream metabolite N-methylhydantoin may reflect alterations in energy homeostasis, muscle metabolism, and mitochondrial efficiency under diabetic conditions. N-methylhydantoin, a product of creatinine metabolism, is ultimately converted to glycine and then pyruvate, which enters the TCA cycle [55]. Although direct links between N-methylhydantoin and obesity or diabetes are not well established, alterations in its levels may reflect changes in creatine metabolism, which is crucial for energy homeostasis. Recent studies have reported altered plasma creatine levels in diabetes patients [56,57]. Elevated extracellular creatine and reduced intracellular phosphorylcreatine-to-creatine ratio may indicate impaired cellular energy status, suggesting mitochondrial dysfunction as a potential mechanism in diabetes pathophysiology [57,58].

Creatine, a nitrogenous acid produced primarily in the liver, kidneys, and pancreas, plays a crucial role in cellular energy metabolism. Its synthesis involves a pathway starting with arginine and glycine, ultimately resulting in creatine formation through the actions of L-arginine:glycine amidinotransferase and N-guanidinoacetate methyltransferase [59]. Furthermore, specific alterations in skeletal muscle creatine metabolism, particularly in the expression of creatine transporter (SLC6A8), have been associated with diabetes risk [58]. The present study revealed distinct patterns in creatine levels across different treatment groups. ZDF rats on the moderate high-fat Diet B treatment showed higher creatine levels, reflecting upregulation or reduced utilisation that maybe linked to altered muscle metabolism in response to a moderate high-fat diet. Conversely, the Diet A and metformin groups exhibited lower levels, which may indicate improved mitochondrial function through increased energy expenditure. In contrast to diabetic rats, Z_lean_ rats receiving both Diet A and Diet B showed significant upregulation of creatine levels compared to controls, indicating that both diets, irrespective of macronutrient formulation could influence creatine metabolism. These findings both align with and diverge with previous studies, highlighting the complex relationship between creatine metabolism and metabolic disorders. For instance, the present study’s results partially corroborate Guan et al. [53] who reported diminished creatine excretion in rats with prolonged diabetes. However, they differ from Hyeon et al. [60] who reported increased urinary creatine levels in diabetic mice and Zhao et al. [54] who observed elevated urinary creatinine levels in obese Zucker and Goto-Kakizaki rats compared to their lean counterparts.

In the present study, N-methylhydantoin levels were significantly elevated with Diet A and B in both ZDF and Z_lean_ rats but lowered with metformin treatment, likely reflecting increased creatine supply from dietary substrates regardless of the diabetes status. Conversely, the lower levels observed in the metformin group could be related to improved mitochondrial function consistent with metformin’s effects on skeletal muscle metabolism [58], which is closely linked to creatine metabolism and energy production. The present study’s findings of elevated N-methylhydantoin levels from Diet A and Diet B treatments, particularly from the high-fat Diet B, align with Hyeon et al. [60], who reported increased urinary N-methylhydantoin in diabetic mice. Pharmacological interventions (metformin in the present study and losartan in theirs) led to lower N-methylhydantoin levels, suggesting potential therapeutic implications for metabolic disorders. However, it is important to note that while the present study analysed plasma N-methylhydantoin, Hyeon et al. [60] examined urinary levels, which may account for differences in observations. Interestingly, Zhang et al. [61] reported reduced faecal N-methylhydantoin in mice fed a high-fat diet compared to those on a normal diet, though the mechanisms remain unclear. Additionally, Yang et al. [62] identified N-methylhydantoin as a predictor of Western diet intake in mice.

### 3.3. Lipid Pathway

The present study revealed that carnitine levels were increased by both Diet A and Diet B in ZDF and Z_lean_ rats compared to controls, with effects in the latter group being significant, whereas metformin treatment led to lower levels. Fatty acid transport and mitochondrial β-oxidation are critical processes in energy production, particularly under high-fat dietary conditions. Carnitine provides important insights into the regulation of lipid utilisation and metabolic flexibility. As a water-soluble amino acid derivative [63], carnitine plays a crucial role in cellular energy production by facilitating the transport of long-chain fatty acids into mitochondria [64] for β-oxidation and regulating both fatty acid oxidation and gluconeogenesis [65]. Carnitine supplementation has been shown to influence glucose metabolism in individuals with diabetes by enhancing glucose absorption [66], storage [67], and oxidation [68]. The importance of carnitine in metabolic health is further underscored by studies linking intracellular accumulation of acyl-CoA derivatives to the development of insulin resistance in skeletal muscle and cardiac tissue [69]. Moreover, research has explored the causal relationship between carnitine deficiency and the development of mitochondrial dysfunction and insulin resistance under chronic metabolic stress, such as obesity [70]. The upregulation of carnitine observed in both ZDF and Z_lean_ rats fed Diets A and B, which was particularly significant in Z_lean_ rats, suggests that improved fatty acid metabolism via increased β-oxidation may be independent of diabetes status, highlighting a direct dietary effect on metabolic pathways. Conversely, the lower carnitine levels in ZDF rats treated with metformin could be attributed to metformin-induced inhibition of mitochondrial complex I, which may alter carnitine utilisation and contribute to improved glucose tolerance [71]. Lower carnitine levels with metformin treatment in our study, although not statistically significant, introduce a potential novel finding that has been scarcely reported in the literature. The present study’s findings partially align with previous reports of elevated carnitine levels in obese mice, particularly those on a high-fat diet [72,73]. However, they contrast with observations of lower plasma carnitine levels in obese control rats compared to lean rats [74]. These discrepancies highlight the complexity of carnitine metabolism across different metabolic states and animal models.

### 3.4. Alternate Amino Acid Pathway

Branched-chain amino acid metabolism, particularly valine catabolism, is of interest because of its role in metabolic regulation and insulin sensitivity. 3-aminoisobutyrate, a key intermediate in this pathway, may serve as an indicator of altered amino acid utilisation and metabolic adaptation in obesity and diabetes. 3-aminoisobutyrate, a product of valine catabolism, is generated through the action of 4-aminobutyrate aminotransaminase, primarily found in the brain, kidneys, liver, and muscles [75,76,77]. This process is bidirectional, with the same enzyme capable of converting 3-aminoisobutyrate back to L-methylmalonyl semialdehyde, which can then be oxidised to propionyl-CoA [78]. Recent studies have linked 3-aminoisobutyrate to diabetes, with Barlow et al. [79] reporting markedly elevated fasting plasma 3-aminoisobutyrate levels in diabetes patients compared to non-diabetic controls, particularly among those with decreased insulin secretion. The present study observed higher 3-aminoisobutyrate trends related to both diet treatments in ZDF and Z_lean_ rats with significant levels observed in ZDF rats on Diet B while both diets led to significant elevations in lean rats. Elevation of 3-aminoisobutyrate levels after both diets is a likely biological response to feeding, but the fact that this response reached significance only in diabetic rats on Diet B suggests that even a moderate high-fat content may contribute to altered amino acid metabolism in this group, although additional factors cannot be excluded. This elevation in ZDF rats may reflect a compensatory response to the obese and diabetic state in the Diet A group, while the more pronounced increase with the Diet B group could indicate a response to the high-fat diet exposure and potentially impaired insulin secretion or more severe insulin resistance. Conversely, the lower 3-aminoisobutyrate levels observed in the metformin-treated group may suggest improved insulin secretion, aligning with Barlow et al.’s [79] findings in diabetes patients. The present study’s observations regarding 3-aminoisobutyrate levels with the diet and metformin treatment options contrast with other studies. Wu et al. [80] reported lower levels in mice with diabetic kidney disease compared to controls, whereas in the current study reduced 3-aminoisobutyrate levels were observed only with metformin treatment in ZDF rats. Notably, the decrease in 3-aminoisobutyrate associated with metformin treatment found in the present study has not previously been reported in the literature. While this reduction could indicate improved metabolic function, it may also be a consequence of the rats’ altered metabolic state. The precise mechanism and implications of this metformin effect warrant further investigation. The varied findings across studies underscore the potential of 3-aminoisobutyrate as a biomarker for different metabolic states. Gao et al. [81] found significant differences in serum 3-aminoisobutyrate concentrations between spontaneously hypertensive rats and controls, while Sun et al. [82] demonstrated its ability to distinguish between obese and control rats. These observations, combined with the present study’s results, suggest that 3-aminoisobutyrate levels may reflect nuanced metabolic changes in response to various conditions and interventions.

### 3.5. Gut-Mediated Metabolite Pathway

Choline metabolism is closely linked to lipid transport, membrane integrity, and one-carbon metabolism. Alterations in choline levels may indicate disruptions in lipid metabolism and metabolic regulation associated with obesity and insulin resistance. Choline, a vital nutrient obtained from dietary sources or synthesised de novo [83,84], plays crucial roles in cellular structure and function as a component of membrane phospholipids [85]. Its metabolism involves four pathways producing acetylcholine, trimethylamine, betaine, and phospholipids [86]. The complex relationship between choline and metabolic disorders is highlighted by conflicting findings. While choline deficiency in mice reduced plasma glucose levels and enhanced glucose tolerance, it also aggravated fatty liver [87]. In humans, studies have reported mixed results regarding the association between dietary choline intake and diabetes risk or insulin sensitivity [88,89]. In the present study, variations in plasma choline levels were observed across different interventions in ZDF rats. Diet A showed higher levels while Diet B and metformin treatment led to lower levels, although these changes were not statistically significant. In contrast, lower choline levels were observed in Z_lean_ rats receiving Diet A (*p* < 0.05) and Diet B (*p* > 0.05). The higher choline levels observed in ZDF rats receiving Diet A but not Diet B may indicate altered lipid metabolism associated with obesity and diabetes, given choline’s role in lipid transport and metabolism [86]. Conversely, the lower choline levels in the metformin group might suggest improved insulin sensitivity through enhanced glucose uptake [41]. The lower choline levels in Z_lean_ rats further highlight the complexity of choline metabolism across different metabolic states. These findings suggest that choline metabolism may be influenced by factors beyond obesity and diabetes, potentially including genetic or physiological differences between ZDF and Z_lean_ rats. The present study’s findings partially align with previous studies in observing altered choline levels in obesity and diabetes, but contrast in the direction of changes. Earlier studies consistently reported elevated urinary choline levels in obese and obese-diabetic rat models compared to normal controls, with this trend persisting even with metformin treatment [54,90]. The discrepancies between the present study and previous findings could be attributed to differences in biological matrices (plasma versus urine), diet composition, the severity and duration of obesity and diabetes in the animal model used.

### 3.6. Intermediary Energy Metabolism

The Krebs cycle is a central hub of cellular energy metabolism, integrating inputs from carbohydrates, lipids, and amino acids. Citrate, as a key intermediate, reflects shifts in energy production and lipid role in energy metabolism and lipid synthesis. Synthesised in mitochondria, excess citrate is exported to the cytoplasm [91] where it can be converted to acetyl-CoA [92], a precursor for lipid and cholesterol synthesis [91,93,94]. In mice, elevated citrate levels have been associated with impaired glucose tolerance and increased inflammation, contributing to insulin resistance [91]. In the present study, ZDF rats experienced non-significant elevations in plasma citrate when subjected to Diets A and B, whereas a decreasing trend was observed with metformin treatment whilst diet treatments in Z_lean_ rats also caused significantly lower citrate levels. The higher citrate levels among ZDF rats receiving dietary treatments might indicate increased lipid synthesis through enhanced conversion to acetyl-CoA [91,93,94], whereas the lower levels could reflect improved insulin sensitivity in the metformin group through enhanced glucose uptake, potentially mediated by increases in glucose transporter GLUT4 activity [41]. The lower citrate levels in Z_lean_ rats receiving the same diets add complexity to the interpretation of citrate changes and suggest that citrate metabolism may be influenced by a combination of metabolic state, genetic factors and possibly other physiological differences between ZDF and Z_lean_ rats. In rodent models of diabetes and obesity lower urinary citrate levels have been reported in obese or diabetic rats compared to normal rats [36,90] or controls [70], with metformin treatment further reducing citrate concentrations in obese diabetic rats [90]. However, rats with prolonged diabetes (15 weeks) were noted with elevated urinary citrate levels [53]. The present study aligns with other studies noting metformin treatment of diabetic rats caused lower citrate levels [36,52,90]. It is important to note that the present study analysed plasma metabolite levels, whereas prior studies primarily investigated urinary concentrations, which may contribute to the contrasting trends observed.

### 3.7. Glucose Metabolism, Energy Homeostasis and Diabetes

Metabolites such as 1,6-anhydro-β-D-glucose provide insight into alterations in glucose utilisation and potential dysregulation of glycolytic and related pathways. This metabolite became elevated in ZDF rats receiving Diet A and B treatments but contrarily became lower with metformin treatment or in Z_lean_ rats with the effect significantly pronounced with the moderate high-fat Diet B. While 1,6-anhydro-β-D-glucose’s direct link to obesity and diabetes has not been extensively studied, its potential to impact blood glucose levels and insulin sensitivity warrants investigation [95] as it is directly phosphorylated to glucose-6-phosphate [96]. The higher levels observed in the diabetic rats receiving dietary treatments may indicate a disruption in glucose metabolism, potentially exacerbating insulin resistance whilst the lower levels seen in the metformin group suggests an improvement in glucose metabolism and insulin sensitivity, aligning with metformin’s known effects [40]. However, the lower 1,6-anhydro-β-D-glucose levels observed in Z_lean_ rats receiving Diet A and Diet B add complexity to these findings. This suggests that the relationship between 1,6-anhydro-β-D-glucose and glucose metabolism may be influenced by factors beyond insulin resistance or metformin treatment, including genetic or physiological differences between ZDF and Z_lean_ rats. The present study’s results partially align with recent studies on 1,6-anhydro-β-D-glucose in rodent models, which has shown alterations in its levels with various interventions. For instance, resveratrol treatment significantly lowered 1,6-anhydro-D-glucose in rats’ heart and liver tissues [97] and liraglutide treatment reduced levels in obese rats [98]. Interestingly, metformin-associated changes observed in the present study mirrored these findings, although the changes were not statistically significant.

## 4. Materials and Methods

### 4.1. Animal Experiment

Ten-week-old male ZDF rats (*n* = 24) and Z_lean_ (*n* = 6) rats were purchased from Vital River, Beijing, China. The Z_lean_ rats served as a control group, allowing for the comparison of dietary effects in a non-obese, non-diabetic model. All rats were quarantined for two weeks to acclimatise to the experimental conditions (room temperature of 19–24 °C, 40–60% humidity, 12/12 h light/dark cycle). During the first week, the rats were provided with a standard rodent maintenance diet (11% fat, 65% carbohydrate and 24% protein from total energy in kcal) (Altromin GmbH & Co. KG, Lage, Germany) and distilled water *ad libitum*. Following the acclimatisation period, fasting blood glucose levels were measured and levels exceeding 200 mg/dL were considered indicative of diabetes [99] confirming the diabetic status of the ZDF rats before the experiment began.

ZDF rats were then randomly assigned to four treatment groups: control (*n* = 6), Diet A (*n* = 6), Diet B (*n* = 6) and standard diet with metformin (200 mg/kg, *n* = 6) treatments. During the experimental period, one rat in the Diet A group and one rat in the metformin group died, resulting in final sample sizes (*n* = 5) for both Diet A and metformin groups. In parallel, Z_lean_ rats were fed either Diet A (*n* = 3) and Diet B (*n* = 3) to assess the comparative effects of these diets in lean rats. The modified Diet A (Altromin GmbH & Co. KG, Lage, Germany) and Diet B (Altromin GmbH & Co. KG, Lage, Germany) are summarised in Table 6. Sample size determination was described previously [100].

These diets were prepared by the manufacturer to comply to stringent quality control standards. Key ingredients for both diets include corn starch, anhydrous milk fats, casein, maltodextrin, sucrose, soybean oil, α-cellulose, vitamin, mineral, L-cystine, choline bitartrate and tert-butylhydroquinone. Diet A was formulated to achieve 54% carbohydrate, 32% fat, and 14% protein expressed as % of total energy to reflect a typical human diet based on the Malaysian Dietary Guidelines 2020 [101] recommendations. In contrast, Diet B was formulated to reflect a moderate high-fat diet comprising 49% carbohydrate, 37% fat, 14% protein expressed as % of total energy. Although this fat contribution is lower than that of classical high-fat diets (often ≥45–60% of energy), it was selected to increase dietary fat load while maintaining palatability and avoiding extreme weight gain in this protocol, thereby allowing the detection of more graded phenotypic and metabolomic responses in ZDF and Z_lean_ rats. The diets were administered to rats over an eight-week period. At the end of the experiment, the rats were euthanised via cardiac puncture under 5% isoflurane anaesthesia. Blood samples were then collected into lithium heparin tubes and stored at −80 °C for subsequent metabolomics analysis. Throughout the experiment, parameters including general behaviour, body weight changes, food and water intake, and fasting blood glucose levels were monitored and recorded.

### 4.2. Metabolomics Analysis

#### 4.2.1. Sample Preparation

**Figure 12:**
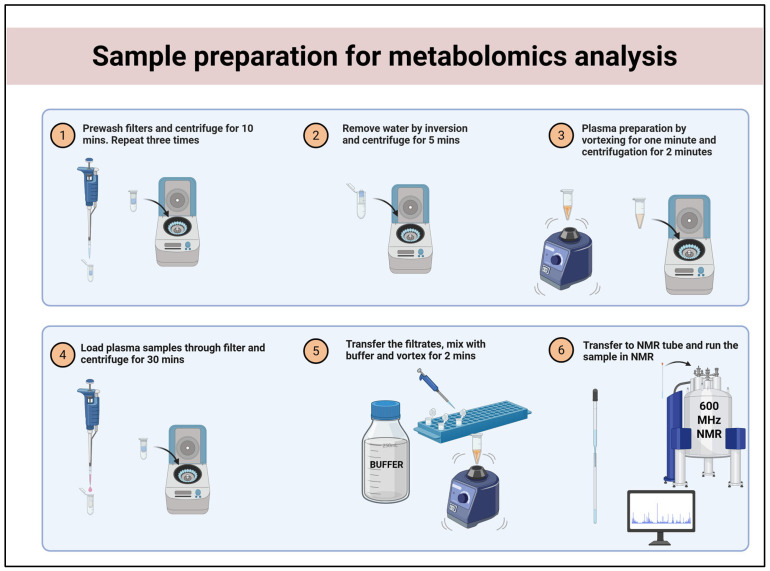
Overview of sample preparation for metabolomics analysis.

The detailed protocol for this study has been published elsewhere [100]. Briefly, frozen plasma samples (400 μL) were thawed and vortexed for 1 min and centrifuged at 10,000 rpm for 2 min to remove solid debris. The resulting plasma supernatant was then filtered through a 0.5 mL, 3 kDa centrifugal filter at 13,800 rpm for 30 min to eliminate macromolecules such as lipids and proteins [102,103]. Prior to filtration, the filters were pre-washed three times with deionised water to remove glycerol and preservative. Any residual water was removed by inverting the filters and centrifuging at 13,800 rpm for five minutes. The filtered plasma samples were transferred to a new tube and diluted with phosphate buffer (KH_2_PO_4_) in deuterium oxide (D_2_O) containing 0.1% 3-(Trimethylsilyl) propionic-2,2,3,3-d_4_ (TSP) and 0.1% imidazole in a 1:2 ratio [104]. Finally, 600 μL of each prepared sample was placed into a 5 mm NMR tube for analysis. The sample preparation is shown (Figure 12).

#### 4.2.2. Spectra Acquisition

The 1D ^1^H-NMR spectra acquired at 26 °C using a JNM-ECZ-600 R 600 MHz spectrometer (JEOL, Tokyo, Japan). Prior to NMR data acquisition, gradient shimming was performed, and D_2_O was used as the internal lock. The combination of presaturation and the Carr-Purcell-Meiboom-Gill (CPMG) pulse sequence was applied to suppress water signals and broad protein resonances. NMR spectra with a spectral width of 12 ppm were acquired using 64 scans and total acquisition time of 26 min [22]. After acquisition, the spectra were processed using the Chenomx NMR suite software, version 9.2 (Chenomx Inc., Edmonton, AB, Canada). The comprehensive acquisition parameters have already been reported previously [100].

Processed NMR spectra were analysed using SIMCA-P software version 17 (Sartorius Stedim Data Analytics AB, Göttingen, Germany) for multivariate data analysis (MVDA). Initially, the data were mean-centred and Pareto-scaled by dividing each variable by the square root of its standard deviation. PCA was performed as an unsupervised technique to visualise dominant clustering tendencies and detect outliers. PLS-DA, a supervised extension of PCA, was applied to maximise class separation using X and Y variables with predefined group identifications. Outliers were detected using Hotelling’s *T*^2^, a multivariate generalisation of the 95% confidence interval. The validity of the PLS-DA models was assessed using CV-ANOVA, complemented by permutation testing (200 permutations) of class labels to evaluate whether the observed discrimination exceeded that expected under random group assignment [105]. Potential biomarkers in PLS-DA were identified as significant spectral regions using loading plots and by assessing VIP values greater than one, along with S-plots. Complementary analyses in R (v4.6.0) calculated group centroids (PCA and PLS-DA) via multivariate ANOVA and visualised 95% confidence ellipses using the ggplot2 (v4.0.3), dplyr (v1.2.1) and mixOmics (v6.36.0) packages, addressing limitations in SIMCA-P’s native visualisation capabilities for centroid uncertainty. Spectral binning provides a rapid approach to NMR data reduction, but its analytical power is inherently limited when multiple metabolites contribute to a single bin or when metabolite signals span bin boundaries [106].

#### 4.2.3. Metabolite Identification and Quantification

Metabolite identification and quantification were performed using Chenomx NMR Suite version 9.2 (Chenomx Inc., Edmonton, AB, Canada) by analysing specific spectral regions (ppm). This identification process involved comparing peak locations, intensities, and linewidths with the 600 MHz HMDB metabolites library, where the area under each peak reflected the relative concentrations of each metabolite. Both targeted and untargeted NMR metabolomics approaches were employed in the present study. The untargeted approach allowed for the discovery of new biomarkers and metabolic pathways, while targeted approach enabled the precise quantification of selected metabolites. These integrated approaches provided a comprehensive analysis of metabolic changes associated with obesity, pre-diabetes, and diabetes, facilitating the identification of key biomarkers and enhancing understanding of the metabolic landscape.

### 4.3. Statistical Analysis

Statistical analysis was carried out using IBM SPSS Statistics (Version 26). Prior to the statistical analysis, the data were log transformed to normalise distributions. Treatment differences in metabolite levels were calculated based on one-way ANOVA. The post hoc comparison was performed using Tukey-HSD test. To account for false discovery rate, a Benjamini–Hochberg correction method was applied with a threshold of 10% FDR and ranked the metabolites according to their *p*-values to identify statistically significant differences across various diet group [107,108]. Graphical representations of metabolite differences were generated using GraphPad Prism (Version 9.5.0; GraphPad Software, San Diego, CA, USA).

### 4.4. Pathway Analysis

Metabolic pathway identification was conducted using the Kyoto Encyclopaedia of Genes and Genomes (KEGG) database through the MetaboAnalyst web application. MetaboAnalyst 6.0 (http://www.metaboanalyst.ca/) is a comprehensive tool for metabolomics data analysis that currently provides access to 1,600 pathways and visualisations for 21 model species with rat (*Rattus norvegicus*) selected for this study [109,110]. The “Metabolic pathway analysis” module was used to explore pathways related to biomarkers associated with diabetes and obesity [111]. Metabolic pathways were visualised as circles on a pathway impact plot, where the *x*-axis represents pathway impact (topology-based impact score) and the *y*-axis represents −log10(*p*) from the enrichment analysis. The size of each circle is proportional to the pathway impact score, such that larger circles indicate more strongly perturbed pathways. The colour of the circles ranges from yellow to red, with darker (redder) colours corresponding to higher statistical significance (lower *p*-values). Pathways represented by large, dark-red circles in the upper-right region of the plot are therefore both highly impacted and highly significant.

## 5. Conclusions

The present study suggests that even modest changes in dietary macronutrient composition can produce substantial, coordinated shifts in multiple metabolic pathways in both ZDF and Z_lean_ rats. Across ketogenic, pyruvate, lipid, amino acid, gut-derived and intermediary energy pathways, Diet A (normal human-like) and Diet B (moderate high-fat) consistently altered key metabolites (3-hydroxybutyrate, 2-hydroxyvalerate, acetate, creatine, N-methylhydantoin, carnitine, 3-aminoisobutyrate, choline, citrate and 1,6-anhydro-β-D-glucose), indicating that diet on its own may have the potential to modify substrate utilisation and mitochondrial energy handling, irrespective of diabetes status. Metformin, in turn, broadly attenuated many of these diet-induced changes, particularly lowering ketone-related and amino acid-derived metabolites, thereby consistent with its role as a systemic modulator of insulin sensitivity, mitochondrial function and glucose utilisation rather than merely a glucose-lowering agent. More specifically, elevation of ketone bodies (3-hydroxybutyrate, 2-hydroxyvalerate) and short-chain fatty acids (acetate) with dietary interventions indicate a shift toward increased fat oxidation and alternative fuel use when carbohydrate availability is relatively low, with Diet B tending to accentuate these changes in ZDF rats. Parallel alterations in creatine and N-methylhydantoin highlight the sensitivity of the creatine–phosphocreatine buffer and pyruvate-linked metabolism to both diet and metformin, pointing to mitochondrial energy status as a central node in diabetes pathophysiology. Changes in carnitine and 3-aminoisobutyrate underscore that fatty acid transport and branched-chain amino acid catabolism are strongly diet-responsive in these models and not confined to diabetic states, while the divergent patterns of citrate, choline and 1,6-anhydro-β-D-glucose between ZDF and Z_lean_ rats emphasise the importance of genetic and physiological background in shaping metabolic responses. Together, these findings outline a mechanistic framework in which normal and moderate high-fat diets reconfigure multiple interconnected pathways (ketogenesis, mitochondrial metabolism, amino acid metabolism, gut-mediated metabolites and glucose handling), and metformin partially normalises these changes, consistent with improved insulin sensitivity.

At the same time, the study has important limitations that frame the next steps. Measurements were restricted to plasma metabolites at a single time point, without direct assessment of tissue-level fluxes, enzyme activities, transporter expression or microbiota composition. In addition, group comparisons were based on parametric ANOVA with Tukey’s HSD post hoc tests, which assume normality and homoscedasticity. Given the small sample sizes and the skewed distribution typical of metabolomics data, non-parametric approaches such as the Kruskal–Wallis test may be more appropriate in future studies to confirm these findings. Non-significant trends in some metabolites may reflect sample size or biological variability rather than an absence of effect, and the use of metformin as the sole pharmacological comparator limits insight into how other antidiabetic agents might differentially modulate these pathways. These constraints do not undermine the observed patterns, but they do mean that the mechanisms proposed here should be viewed as biologically plausible rather than definitively proven.

Building on these results, several future research directions emerge clearly. First, longitudinal and tissue-resolved metabolomics in liver, skeletal muscle, adipose tissue and gut, combined with direct measures of mitochondrial respiration, ketone and acetate flux and insulin signalling, are needed to link the plasma signatures observed here to concrete cellular mechanisms. Second, targeted studies of key nodes highlighted by this work, such as the creatine transporter (SLC6A8), GLUT4, carnitine transport and acyl-CoA handling, and enzymes in 3-aminoisobutyrate and N-methylhydantoin pathways, would clarify how diet and metformin rewire specific metabolic circuits. Third, expanding pharmacological comparisons to include other oral hypoglycaemic agents (e.g., acarbose, sulfonylureas, DPP-4 inhibitors, GLP-1 receptor agonists) will help determine whether the metabolite patterns identified are unique to metformin or represent shared or complementary therapeutic mechanisms. Finally, translational studies in humans, integrating plasma metabolomics with clinical phenotypes of obesity, insulin resistance and T2DM, will be essential to evaluate whether metabolites such as 3-hydroxybutyrate, N-methylhydantoin, 3-aminoisobutyrate, carnitine, choline, citrate and 1,6-anhydro-β-D-glucose might serve as practical biomarkers to guide personalised dietary and pharmacological strategies.

## Figures and Tables

**Table 1:** Concentration of metabolites.

Metabolites	ZDF Control (*n* = 6)	ZDF Diet A (*n* = 5)	ZDF Diet B (*n* = 6)	ZDF Metformin (*n* = 5)	Z_lean_ Diet A (*n* = 3)	Z_lean_ Diet B (*n* = 3)	*p*
1,6-Anhydro-β-D-glucose	3.16 ± 0.09	3.20 ± 0.09	3.32 ± 0.21	3.10 ± 0.06	3.14 ± 0.11	3.02 ± 0.08	0.029 *
2-Hydroxyvalerate	1.26 ± 0.34	1.31 ± 0.13	1.46 ± 0.20	1.14 ± 0.34	0.78 ± 0.07	1.3 ± 0.36	0.040 *
2-Methylglutarate	1.35 ± 0.05	1.30 ± 0.08	1.37 ± 0.06	1.30 ± 0.12	1.31 ± 0.08	1.33 ± 0.11	0.683
2-Oxoglutarate	2.12 ± 0.09	2.15 ± 0.06	2.19 ± 0.10	2.10 ± 0.08	2.14 ± 0.06	2.04 ± 0.14	0.263
3-Aminoisobutyrate	2.47 ± 0.19	2.57 ± 0.10	2.97 ± 0.21	2.38 ± 0.15	2.75 ± 0.12	2.99 ± 0.03	<0.001 *
3-Hydroxybutyrate	2.41 ± 0.19	2.52 ± 0.10	2.92 ± 0.19	2.33 ± 0.13	2.7 ± 0.12	2.94 ± 0.04	<0.001 *
Acetate	2.38 ± 0.06	2.36 ± 0.10	2.49 ± 0.06	2.47 ± 0.07	2.28 ± 0.01	2.38 ± 0.05	0.002 *
Agmatine	1.77 ± 0.18	1.76 ± 0.18	1.76 ± 0.22	1.72 ± 0.18	1.49 ± 0.16	1.97 ± 0.18	0.111
Alanine	2.59 ± 0.12	2.62 ± 0.09	2.49 ± 0.14	2.50 ± 0.08	2.6 ± 0.12	2.62 ± 0.1	0.261
Anserine	2.94 ± 0.02	2.93 ± 0.01	2.96 ± 0.12	2.71 ± 0.21	2.8 ± 0.22	2.95 ± 0.06	0.170
Cadaverine	2.24 ± 0.16	2.23 ± 0.11	2.27 ± 0.12	2.32 ± 0.13	2.31 ± 0.17	2.41 ± 0	0.422
Carnitine	2.21 ± 0.13	2.28 ± 0.06	2.38 ± 0.16	2.15 ± 0.12	2.41 ± 0.15	2.55 ± 0.2	0.006 *
Choline	1.29 ± 0.18	1.43 ± 0.17	1.20 ± 0.22	1.12 ± 0.08	0.98 ± 0.11	1.13 ± 0.27	0.028 *
Citrate	2.44 ± 0.03	2.49 ± 0.09	2.46 ± 0.05	2.41 ± 0.08	2.32 ± 0.06	2.36 ± 0.03	0.010 *
Creatine	2.12 ± 0.07	2.04 ± 0.08	2.17 ± 0.09	2.03 ± 0.12	2.32 ± 0.06	2.44 ± 0.15	<0.001 *
Formate	1.93 ± 0.09	1.87 ± 0.12	1.82 ± 0.06	1.90 ± 0.06	1.77 ± 0.05	1.9 ± 0.07	0.081
Gluconate	3.40 ± 0.24	3.43 ± 0.15	3.28 ± 0.09	3.42 ± 0.14	3.36 ± 0.05	3.16 ± 0.11	0.181
Glucose	4.16 ± 0.08	4.20 ± 0.10	4.16 ± 0.07	4.12 ± 0.14	3.99 ± 0.02	4.07 ± 0.17	0.112
Glutamate	2.07 ± 0.18	2.09 ± 0.17	2.12 ± 0.07	2.04 ± 0.14	2.08 ± 0.16	2.08 ± 0.15	0.952
Glutamine	2.64 ± 0.17	2.75 ± 0.04	2.71 ± 0.09	2.60 ± 0.03	2.69 ± 0.08	2.68 ± 0.08	0.208
Glycine	2.45 ± 0.17	2.57 ± 0.12	2.47 ± 0.12	2.48 ± 0.23	2.41 ± 0.18	2.19 ± 0.07	0.083
Glyclyproline	3.04 ± 0.39	2.99 ± 0.42	2.99 ± 0.15	2.81 ± 0.21	2.94 ± 0.09	2.93 ± 0.15	0.848
Histidine	2.33 ± 0.25	2.35 ± 0.11	2.37 ± 0.09	2.29 ± 0.16	2.17 ± 0.09	2.34 ± 0.32	0.714
Homocysteine	2.81 ± 0.19	2.82 ± 0.15	2.88 ± 0.07	2.69 ± 0.09	2.82 ± 0.09	2.83 ± 0.04	0.251
Hydroxyacetone	1.58 ± 0.03	1.59 ± 0.05	1.59 ± 0.12	1.46 ± 0.07	1.54 ± 0.08	1.46 ± 0.22	0.178
Isoleucine	1.94 ± 0.08	1.92 ± 0.08	1.89 ± 0.12	1.91 ± 0.13	1.85 ± 0.03	1.86 ± 0.06	0.742
Lactate	3.62 ± 0.20	3.71 ± 0.20	3.32 ± 0.20	3.51 ± 0.16	3.3 ± 0.19	3.38 ± 0.35	0.042 *
Leucine	2.06 ± 0.10	2.04 ± 0.10	1.98 ± 0.14	1.97 ± 0.07	1.97 ± 0.04	2.04 ± 0.06	0.574
Lysine	2.52 ± 0.26	2.57 ± 0.17	2.55 ± 0.16	2.39 ± 0.19	2.65 ± 0.15	2.55 ± 0.2	0.542
Methionine	1.98 ± 0.06	2.04 ± 0.03	2.04 ± 0.16	1.87 ± 0.07	2.01 ± 0.08	2.02 ± 0	0.060
N-Methylhydantoin	1.08 ± 0.07	1.09 ± 0.10	1.19 ± 0.05	0.95 ± 0.05	1.29 ± 0.09	1.21 ± 0.04	<0.001 *
Ornithine	2.66 ± 0.32	2.62 ± 0.22	2.47 ± 0.10	2.44 ± 0.13	2.56 ± 0.04	2.62 ± 0.19	0.448
Proline	2.18 ± 0.29	2.04 ± 0.14	1.95 ± 0.09	2.27 ± 0.28	2.12 ± 0.08	2.23 ± 0.27	0.201
Putrescine	1.22 ± 0.21	1.21 ± 0.08	1.17 ± 0.17	1.20 ± 0.19	0.98 ± 0.18	1.4 ± 0.19	0.159
Pyruvate	2.25 ± 0.16	2.34 ± 0.08	2.22 ± 0.08	2.23 ± 0.10	2.18 ± 0.24	2.07 ± 0.12	0.187
Succinate	1.79 ± 0.41	1.98 ± 0.41	2.03 ± 0.16	1.63 ± 0.27	1.69 ± 0.06	2.12 ± 0.4	0.197
Taurine	3.61 ± 0.12	3.63 ± 0.15	3.56 ± 0.09	3.54 ± 0.11	3.38 ± 0.05	3.46 ± 0.13	0.056
Threonine	3.68 ± 0.22	3.79 ± 0.19	3.40 ± 0.20	3.56 ± 0.19	3.39 ± 0.19	3.46 ± 0.34	0.057
Trimethylamine N-oxide	2.49 ± 0.09	2.54 ± 0.13	2.53 ± 0.11	2.46 ± 0.11	2.31 ± 0.07	2.41 ± 0.21	0.126
Tyrosine	3.41 ± 0.30	3.31 ± 0.22	3.34 ± 0.12	3.31 ± 0.10	3.56 ± 0.15	3.58 ± 0.04	0.232
Valine	2.18 ± 0.07	2.15 ± 0.09	2.11 ± 0.13	2.13 ± 0.08	2.12 ± 0.03	2.15 ± 0.03	0.812
*myo*-inositol	3.61 ± 0.08	3.63 ± 0.13	3.36 ± 0.28	3.58 ± 0.09	3.47 ± 0.06	3.58 ± 0.09	0.074
*trans*-4-Hydroxy-L-proline	2.70 ± 0.30	2.63 ± 0.23	2.63 ± 0.08	2.43 ± 0.06	2.48 ± 0.11	2.56 ± 0.1	0.222
π-methylhistidine	3.13 ± 0.24	3.24 ± 0.20	3.17 ± 0.25	3.19 ± 0.19	3.33 ± 0	3.25 ± 0.13	0.801

Notes: Metabolites listed in alphabetical order. Values are reported as mean ± SD (µM); * Significantly different at *p* < 0.05 based on ANOVA.

**Table 2:** Benjamini–Hochberg correction FDR.

No	Metabolites	*p*	Rank	Critical Value B–H
1	3-Hydroxybutyrate	<0.001	1	0.009
2	N-Methylhydantoin	<0.001	2	0.018
3	3-Aminoisobutyrate	<0.001	3	0.027
4	Creatine	<0.001	4	0.036
5	Acetate	0.002	5	0.045
6	Carnitine	0.006	6	0.055
7	Citrate	0.01	7	0.064
8	Choline	0.028	8	0.073
9	1,6-Anhydro-β-D-glucose	0.029	9	0.082
10	2-Hydroxyvalerate	0.040	10	0.091
11	Lactate	0.042	11	0.1

**Table 3:** Finalised significant metabolites between ZDF rats and control.

Metabolites	Control (*n* = 6) (95% CI)	Diet A (*n* = 5) (95% CI)	Delta (% Change)	Diet B (*n* = 6) (95% CI)	Delta (% Change)	Metformin (*n* = 5) (95% CI)	Delta (% Change)	*p* All Group *(d)*
3-Hydroxybutyrate	2.41 ± 0.19 (2.21–2.61)	2.52 ± 0.10 (2.39–2.65)	0.11 (4.56)	2.92 ± 0.19 (2.72–3.12)	0.51 * (21.16)	2.33 ± 0.13 (2.17–2.5)	−0.08 (−3.32)	<0.001 (0.75)
N-Methylhydantoin	1.08 ± 0.07 (1.0–1.16)	1.09 ± 0.10 (0.96–1.21)	0.01 (0.93)	1.19 ± 0.05 (1.14–1.24)	0.11 (10.19)	0.95 ± 0.05 (0.89–1.01)	−0.13 (−12.04)	<0.001 (0.74)
3-Aminoisobutyrate	2.47 ± 0.19 (2.27–2.68)	2.57 ± 0.10 (2.44–2.69)	0.1 (4.05)	2.97 ± 0.21 (2.75–3.2)	0.5 * (20.24)	2.38 ± 0.15 (2.2–2.56)	−0.09 (−3.64)	<0.001 (0.74)
Creatine	2.12 ± 0.07 (2.05–2.19)	2.04 ± 0.08 (1.94–2.14)	−0.08 (−3.77)	2.17 ± 0.09 (2.08–2.26)	0.05 (2.36)	2.03 ± 0.12 (1.88–2.18)	−0.09 (−4.25)	<0.001 (0.71)
Acetate	2.38 ± 0.06 (2.31–2.44)	2.36 ± 0.10 (2.24–2.48)	−0.02 (−0.84)	2.49 ± 0.06 (2.43–2.55)	0.11 (4.62)	2.47 ± 0.07 (2.38–2.55)	0.09 (3.78)	0.002 (0.56)
Carnitine	2.21 ± 0.13 (2.07–2.34)	2.28 ± 0.06 (2.21–2.36)	0.07 (3.17)	2.38 ± 0.16 (2.22–2.55)	0.17 (7.69)	2.15 ± 0.12 (2.0–2.31)	−0.06 (−2.71)	0.006 (0.51)
Citrate	2.44 ± 0.03 (2.41–2.47)	2.49 ± 0.09 (2.37–2.6)	0.05 (2.05)	2.46 ± 0.05 (2.41–2.52)	0.02 (0.82)	2.41 ± 0.08 (2.31–2.51)	−0.03 (−1.23)	0.010 (0.47)
Choline	1.29 ± 0.18 (1.10–1.48)	1.43 ± 0.17 (1.23–1.64)	0.14 (10.85)	1.20 ± 0.22 (0.97–1.43)	−0.09 (−6.98)	1.12 ± 0.08 (1.03–1.22)	−0.17 (−13.18)	0.028 (0.41)
1,6-Anhydro-β-D-glucose	3.16 ± 0.09 (3.07–3.25)	3.20 ± 0.09 (3.1–3.31)	0.04 (1.27)	3.32 ± 0.21 (3.1–3.55)	0.16 (5.06)	3.10 ± 0.06 (3.02–3.17)	−0.06 (−1.9)	0.029 (0.41)
2-Hydroxyvalerate	1.26 ± 0.34 (0.91–1.61)	1.31 ± 0.13 (1.15–1.47)	0.05 (3.97)	1.46 ± 0.20 (1.25–1.67)	0.2 (15.87)	1.14 ± 0.34 (0.72–1.56)	−0.12 (−9.52)	0.040 (0.39)

Notes: Metabolites listed according to *p* value for between group comparison; data presented mean ± standard deviation (µM); * Significance to control is at *p* < 0.05 according to post hoc Tukey HSD Cohen’s *d* effect size: negligible (0.2), moderate (~0.5) and large (>0.8).

**Table 4:** Finalised significant metabolites between Z_lean_ groups compared to control.

Metabolites	Control (*n* = 6) (95% CI)	Z_lean_ Diet A (*n* = 3) (95% CI)	Delta (% Change)	Z_lean_ Diet B (*n* = 3) (95% CI)	Delta (% Change)	*p* All Group *(d)*
3-Hydroxybutyrate	2.41 ± 0.19 (2.21–2.61)	2.7 ± 0.12 (2.4–3.0)	0.29 (12.03%)	2.94 ± 0.04 (2.83–3.04)	0.53 (21.99%)	<0.001 (0.75)
N-Methylhydantoin	1.08 ± 0.07 (1.0–1.16)	1.29 ± 0.09 (1.08–1.51)	0.21 (19.44%)	1.21 ± 0.04 (1.12–1.3)	0.13 (12.04%)	<0.001 (0.74)
3-Aminoisobutyrate	2.47 ± 0.19 (2.27–2.68)	2.75 ± 0.12 (2.45–3.04)	0.28 (11.34%)	2.99 ±0.03 (2.91–3.04)	0.52 (21.05%)	<0.001 (0.74)
Creatine	2.12 ± 0.07 (2.05–2.19)	2.32 ± 0.06 (2.18–2.47)	0.2 (9.43%)	2.44 ± 0.15 (2.07–2.8)	0.32 (15.09%)	<0.001 (0.71)
Acetate	2.38 ± 0.06 (2.31–2.44)	2.28 ± 0.01 (2.27–2.3)	−0.1 (−4.2%)	2.38 ± 0.05 (2.24–2.51)	0	0.002 (0.56)
Carnitine	2.21 ± 0.13 (2.07–2.34)	2.41 ± 0.15 (2.04–2.79)	0.2 (9.05%)	2.55 ± 0.2 (2.05–3.05)	0.34 (15.38%)	0.006 (0.51)
Citrate	2.44 ± 0.03 (2.41–2.47)	2.32 ± 0.06 (2.18–2.47)	−0.12 (−4.92%)	2.36 ± 0.03 (2.28–2.44)	−0.08 (−3.28%)	0.010 (0.47)
Choline	1.29 ± 0.18 (1.10–1.48)	0.98 ± 0.11 (0.72–1.24)	−0.31 (−24.03%)	1.13 ± 0.27 (0.45–1.81)	−0.16 (−12.4%)	0.028 (0.41)
1,6-Anhydro-β-D-glucose	3.16 ± 0.09 (3.07–3.25)	3.14 ± 0.11 (2.88–3.41)	−0.02 (−0.63%)	3.02 ± 0.08 (2.82–3.22)	−0.14 (−4.43%)	0.029 (0.41)
2-Hydroxyvalerate	1.26 ± 0.34 (0.91–1.61)	0.78 ± 0.07 (0.59–0.97)	−0.48 (−38.10%)	1.3 ± 0.36 (0.41–2.19)	0.04 (3.17%)	0.040 (0.39)

Notes: Metabolites listed according to *p* between group; data presented mean ± standard deviation (µM); Significance to control is at *p* < 0.05 according to post hoc Tukey HSD Cohen’s *d* effect size: negligible (0.2), moderate (~0.5) and large (>0.8).

**Table 5:** Summary of trends for top-ranked metabolites.

Metabolites	ZDF Diet A	ZDF Diet B	ZDF Metformin	Z_lean_ Diet A	Z_lean_ Diet B
3-Hydroxybutyrate	↑	↑ *	↓	↑ *	↑ *
N-Methylhydantoin	↑	↑	↓	↑ *	↑ *
3-Aminoisobutyrate	↑	↑ *	↓	↑ *	↑ *
Creatine	↓	↑	↓	↑ *	↑ *
Acetate	↓	↑	↑	↓ *	=
Carnitine	↑	↑	↓	↑ *	↑ *
Citrate	↑	↑	↓	↓ *	↓ *
Choline	↑	↓	↓	↓ *	↓
1,6-Anhydro-β-D-glucose	↑	↑	↓	↓	↓ *
2-Hydroxyvalerate	↑	↑	↓	↓ *	↑

Notes: Trends relative to control; ↑ denotes increased trend; ↓ denotes decreased trend; = denotes no change; * Significance at *p* < 0.05 relative to control.

**Table 6:** Nutrient percentage of Diet A and Diet B.

Nutrients	Diet A (Normal Human Diet)	Diet B (Low-Carbohydrate Moderate High-Fat Diet)	Control (Rodent Maintenance Diet)
Carbohydrate	54%	49%	65%
Fat	32%	37%	11%
Protein	14%	14%	24%

Note: expressed as % of total energy.

## Data Availability

The raw data supporting the conclusions of this article will be made available by the authors on reasonable request. A proposal with a detailed description of study objectives and a statistical analysis plan will be needed for assessment of requests. Additional materials might also be required during the process of assessment. Deidentified participant data will be provided after approval by the investigators.

## Supplementary Materials

The following supporting information can be downloaded at https://www.mdpi.com/article/10.3390/ijms27157017/s1.

## Abbreviations

The following abbreviations are used in this manuscript:

ANOVA	analysis of variance
ARIC	Atherosclerosis Risk in Communities
BMI	body mass index
CKD	chronic kidney disease
CPMG	Carr-Purcell-Meiboom-Gill
CV-ANOVA	analysis of variance of cross-validated residuals
CVD	cardiovascular disease
DALY	disability-adjusted life year
FDR	false discovery rate
HMDB	Human Metabolome Database
HSD	Honest Significant Difference
KEGG	Kyoto Encyclopaedia of Genes and Genomes
MANS	Malaysian Adult Nutrition Survey
MLS	Malaysia Lipid Study
MVDA	multivariate data analysis
NCD	non-communicable disease
NHMS	National Health and Morbidity Survey
NMR	nuclear magnetic resonance
OPLS-DA	orthogonal partial least squares-discriminant analysis
PCA	principal component analysis
PLS-DA	partial least squares-discriminant analysis
PURE	Prospective Urban Rural Epidemiology
SSB	sugar-sweetened beverage
T2DM	type 2 diabetes mellitus
TCA	tricarboxylic acid
TSP	3-(Trimethylsilyl) propionic-2,2,3,3-d_4_
VIP	variable importance in projection
ZDF	Zucker Diabetic Fatty
Z_lean_	lean Zucker
