# Supplementary material for: Metabolomic Insights on Obesity and Diabetes from Feeding Diets Varying in Carbohydrate–Fat Ratios in Zucker Diabetic Fatty (ZDF) and Lean Zucker (Zlean) Rats
Source: Int J Mol Sci. 2026 Aug 4;27(15):7017. doi: 10.3390/ijms27157017 (PMC13466821; doi:10.3390/ijms27157017)
Supplement: Supplementary file 1 [file ijms-27-07017-s001.zip › ijms-4443481-supplementary.pdf]

Supplementary Materials

i. Control and Diet A

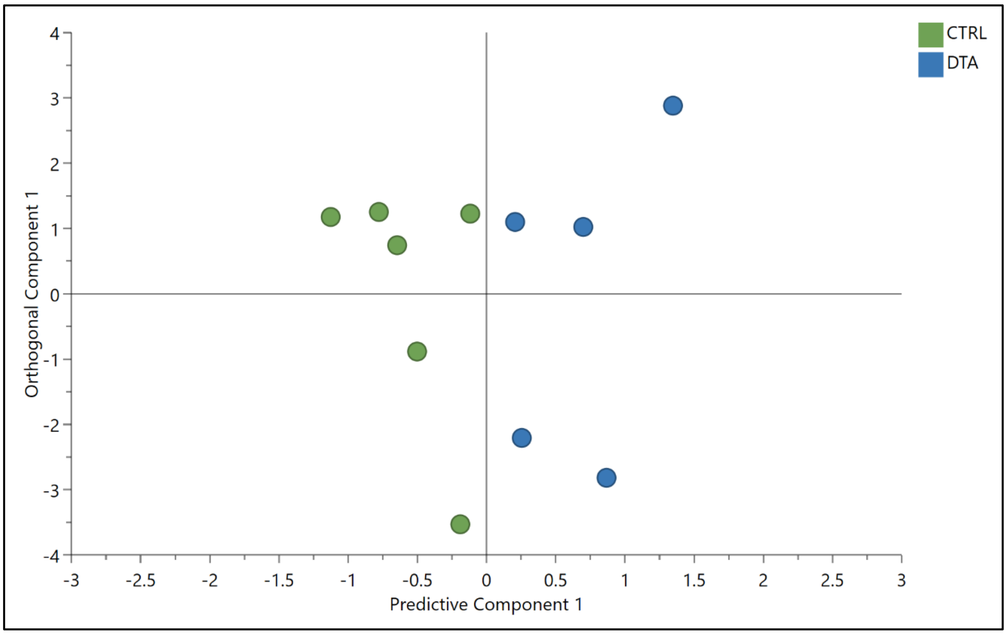

Figure S1. OPLS-DA score plot in ZDF rats between control and Diet A group.

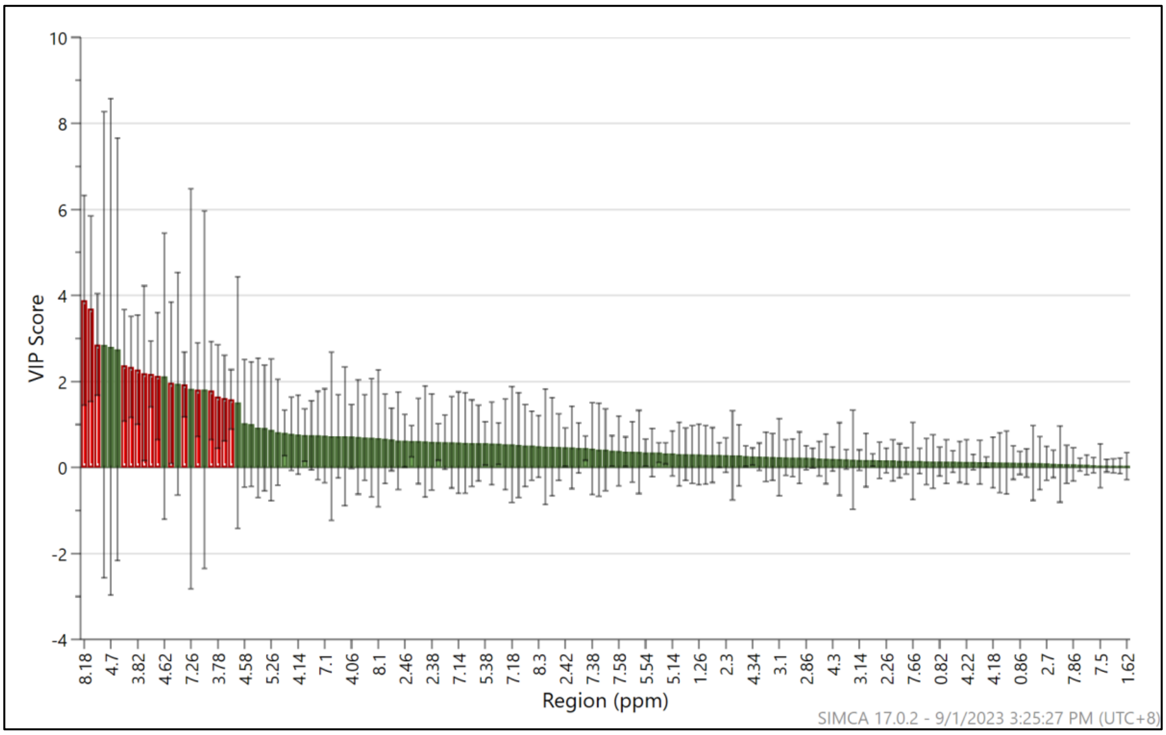

Figure S2. VIP plot for important NMR spectral regions (ppm) highlighted in red between control and Diet A.

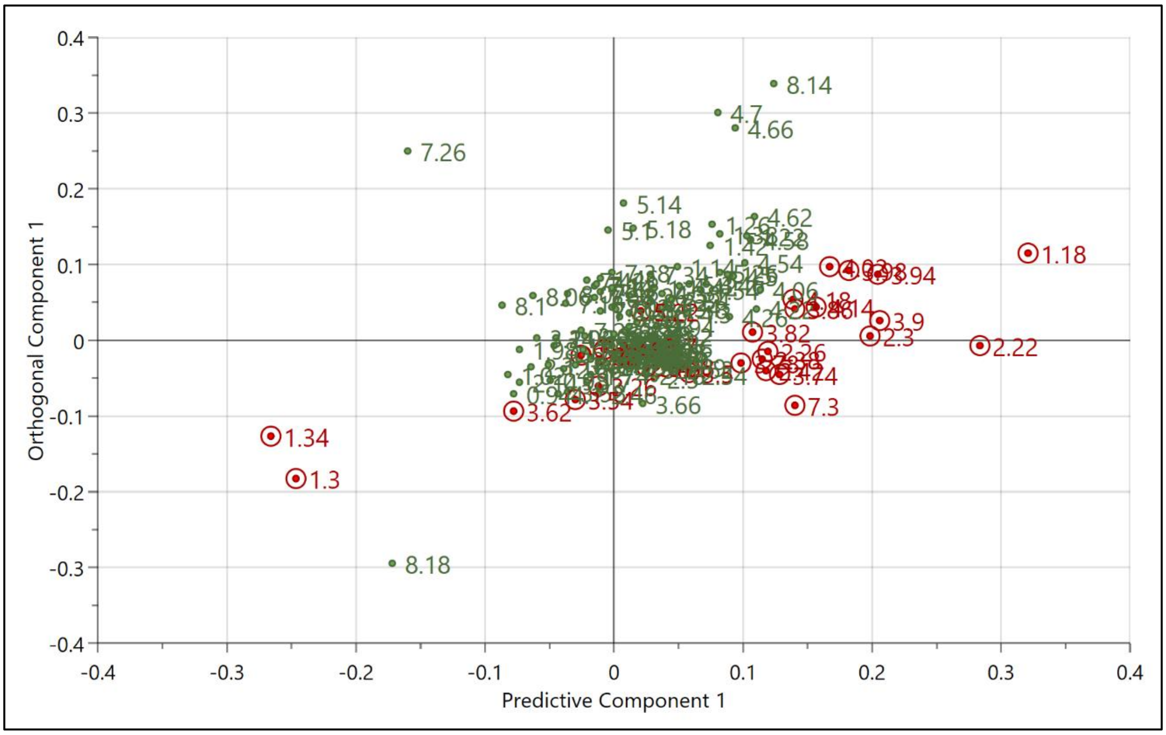

Figure S3. Corresponding loading plot with highlighted NMR spectral regions (ppm) highlighted in red between control and Diet A.

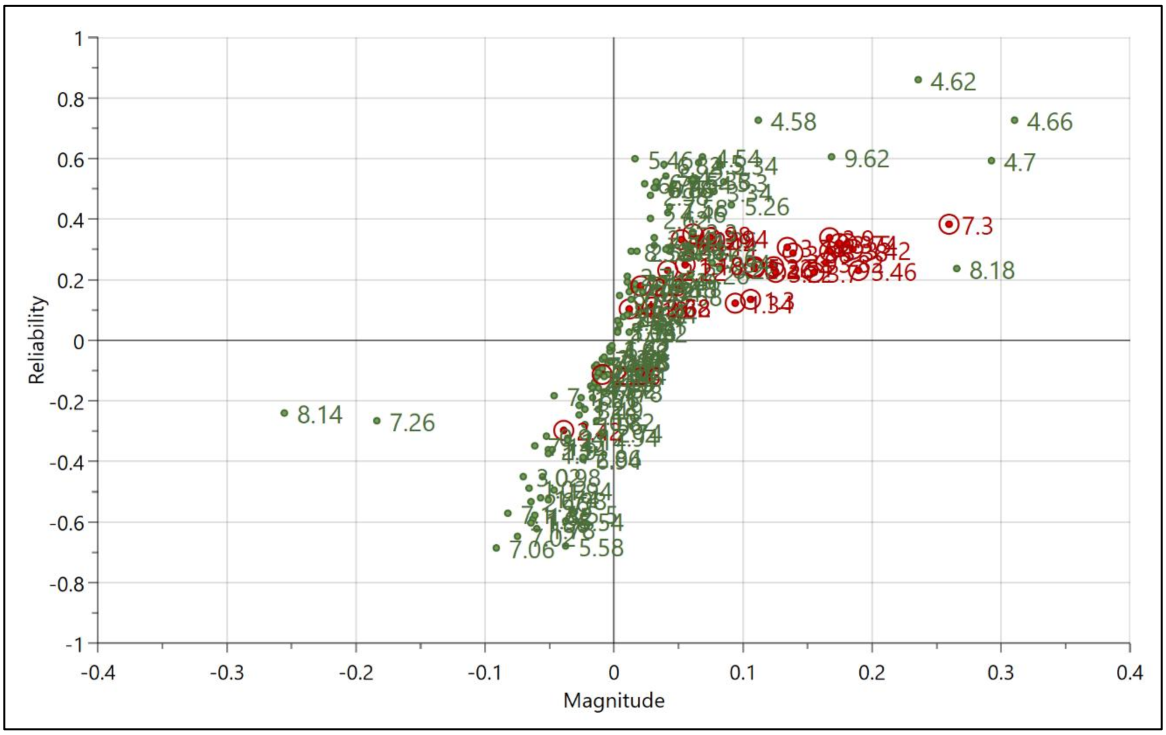

Figure S4. Corresponding S-plot with highlighted NMR spectral regions between control and Diet A.

ii. Control and Diet B

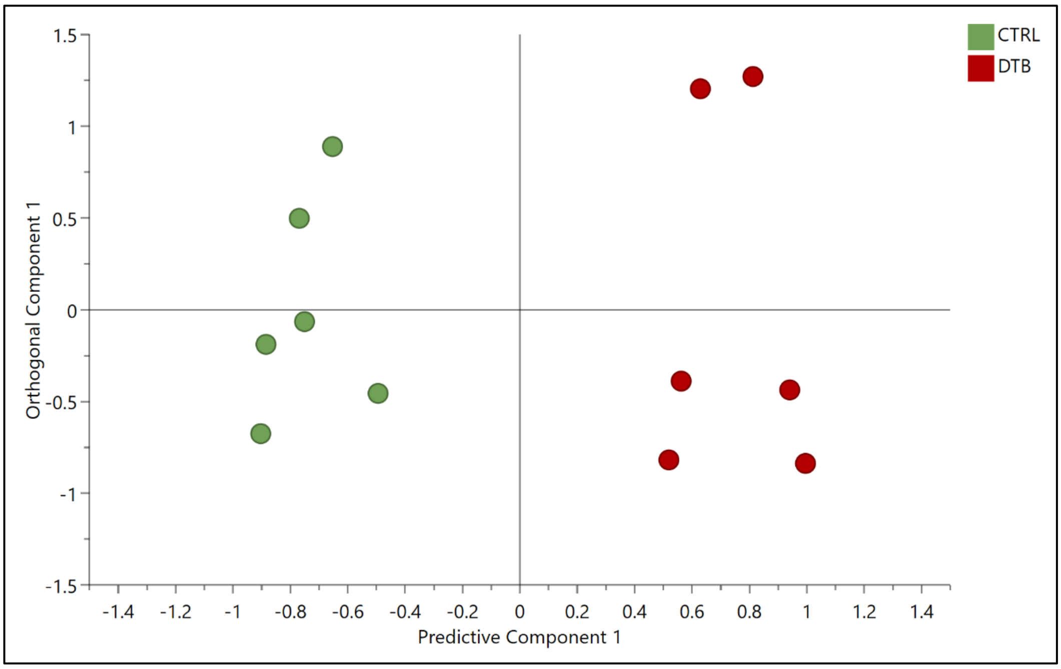

Figure S5. OPLS-DA score plot in ZDF rats between control and Diet B group.

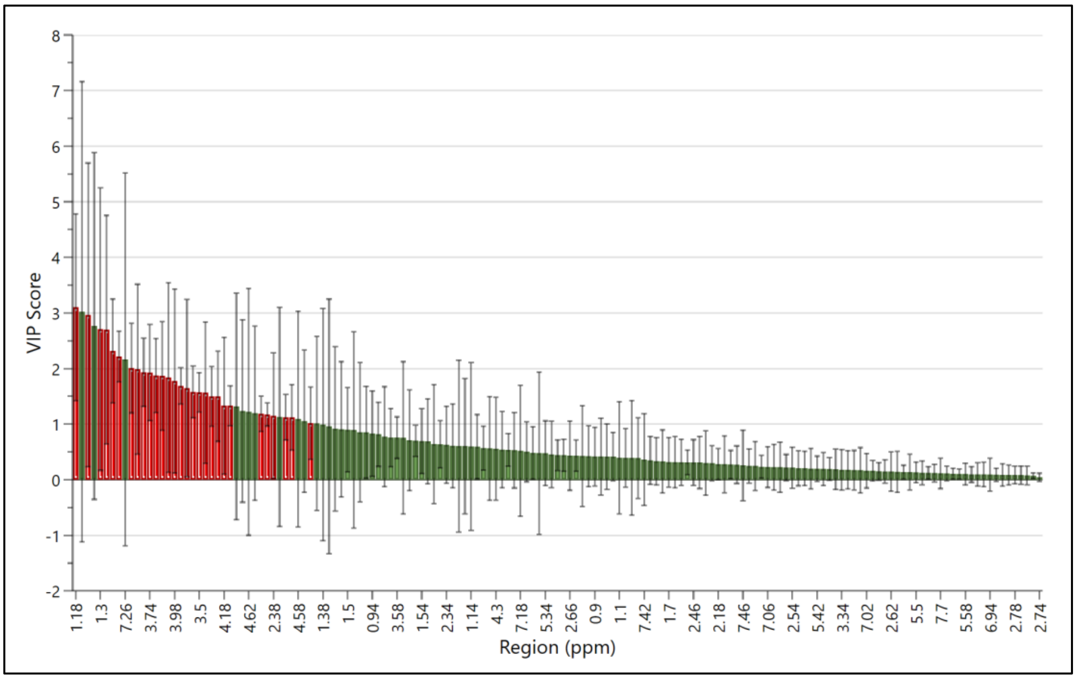

Figure S6. VIP plot for important NMR spectral regions (ppm) highlighted in red between control and Diet B.

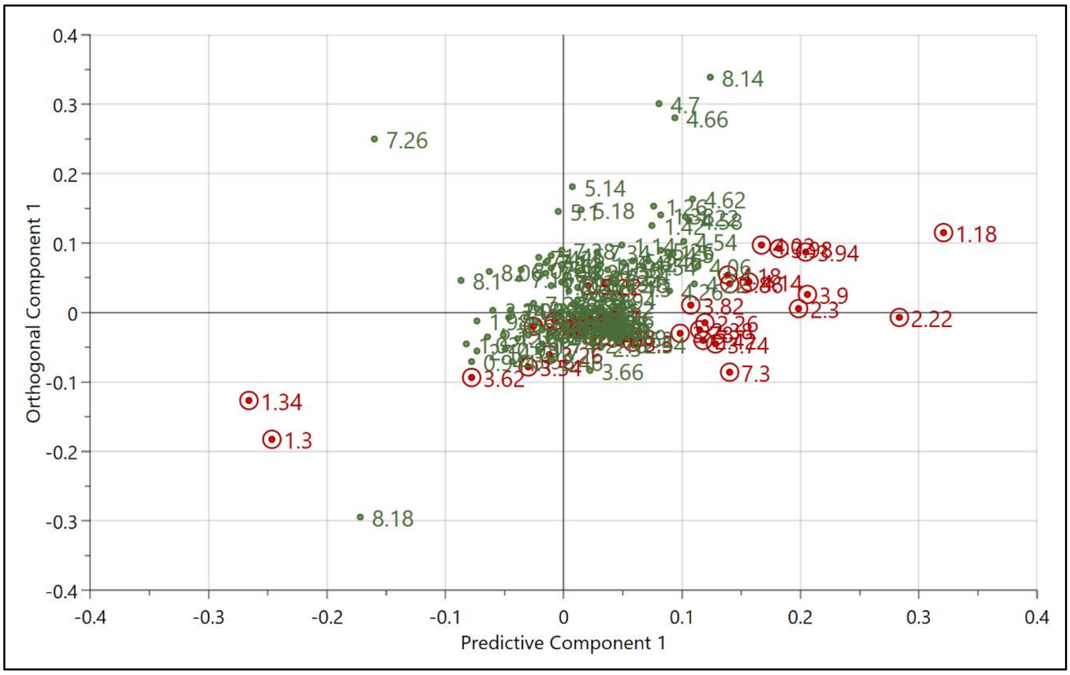

Figure S7. Corresponding loading plot with highlighted NMR spectral regions (ppm) highlighted in red between control and Diet B.

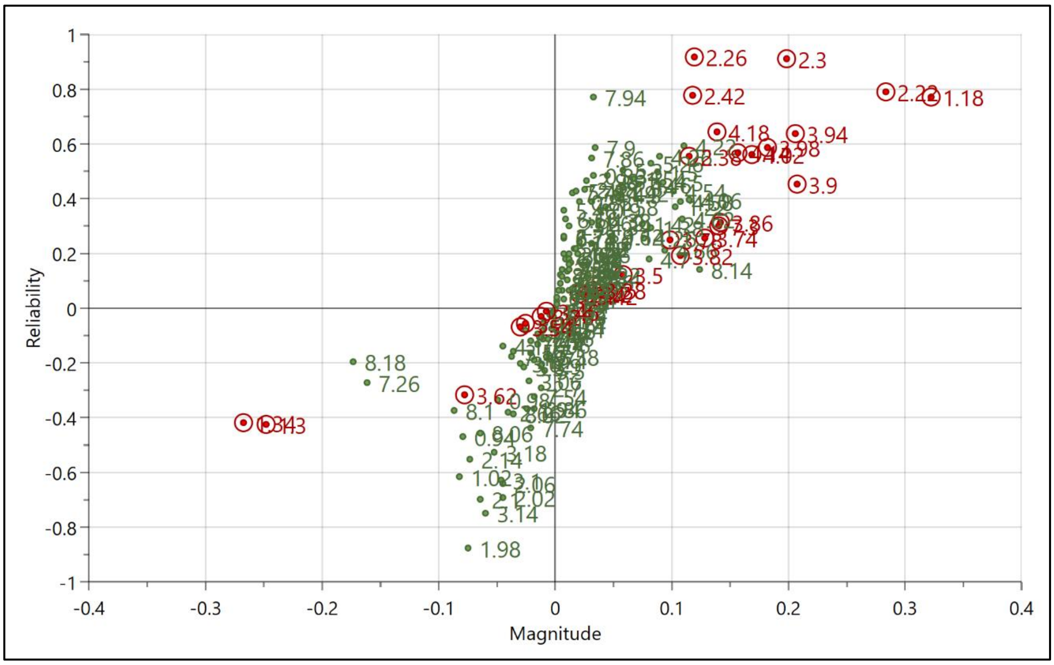

Figure S8. Corresponding S-plot with highlighted NMR spectral regions between control and Diet B.

iii. **Control and Metformin**

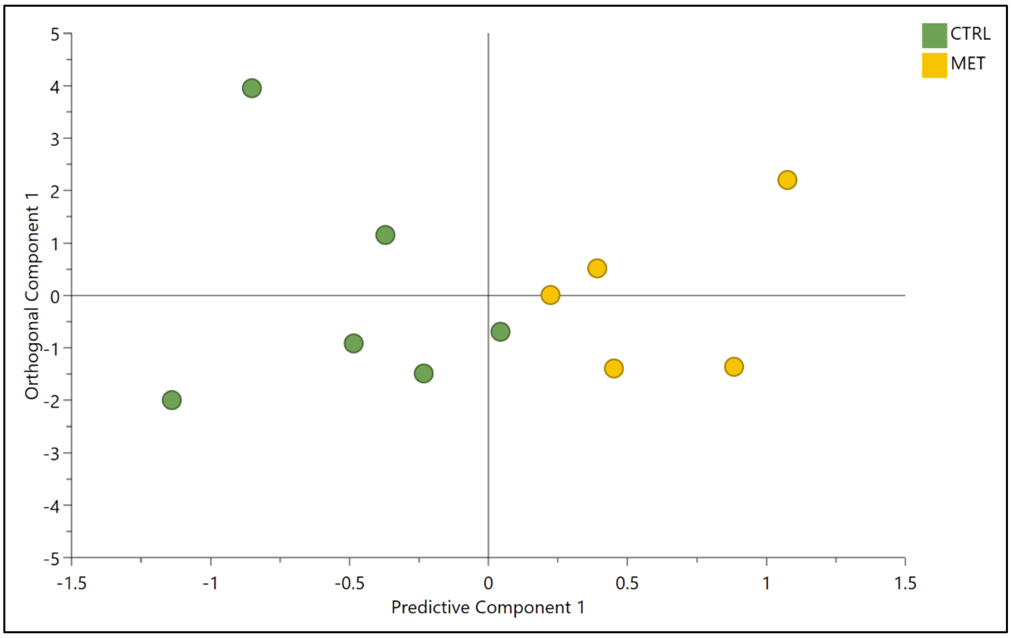

Figure S9. OPLS-DA score plot in ZDF rats between control and metformin group.

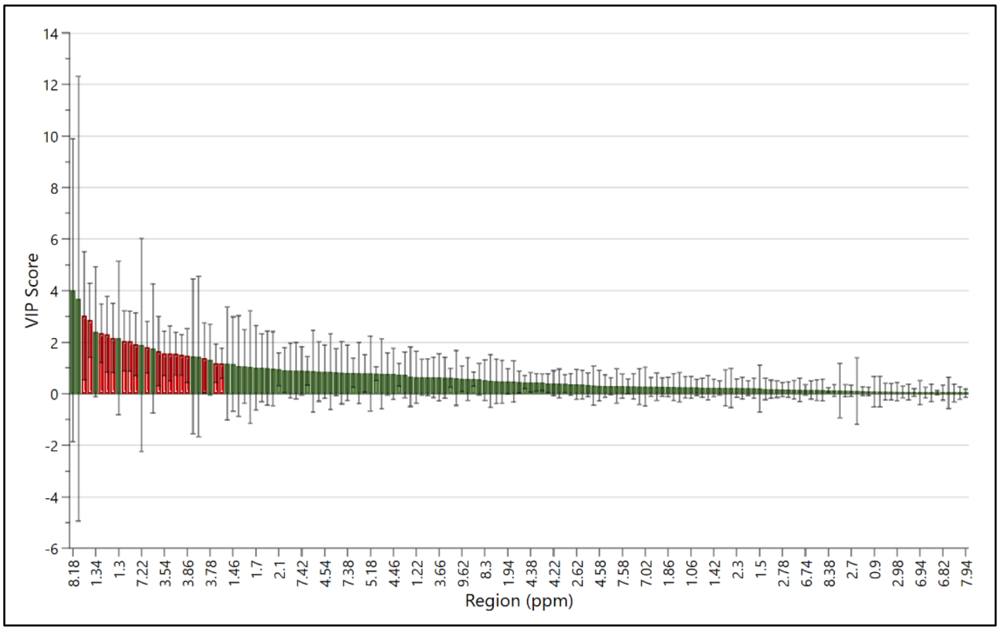

Figure S10. VIP plot for important NMR spectral regions (ppm) highlighted in red between control and metformin.

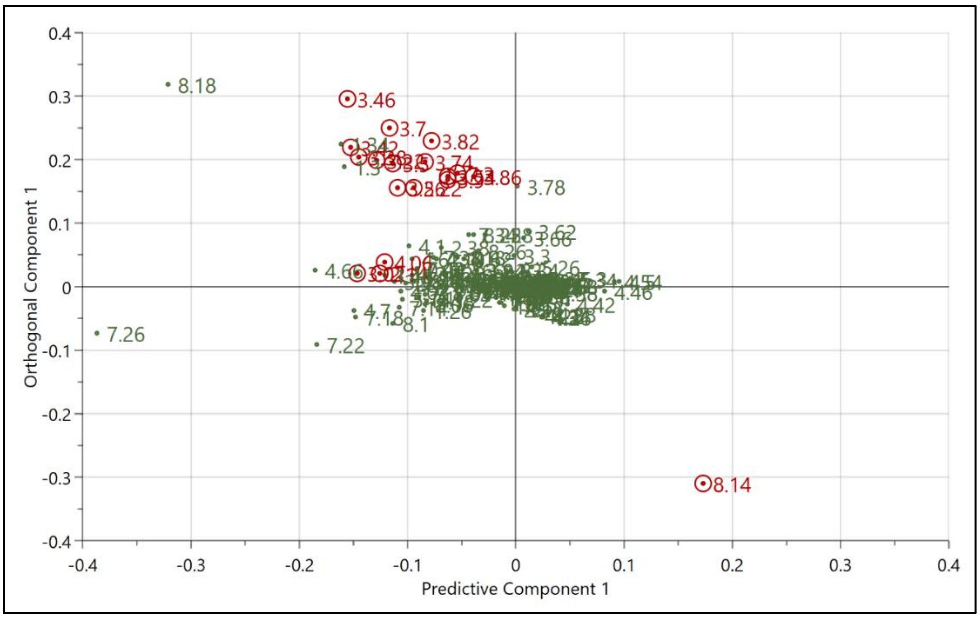

Figure S11. Corresponding loading plot with highlighted NMR spectral regions (ppm) highlighted in red between control and metformin.

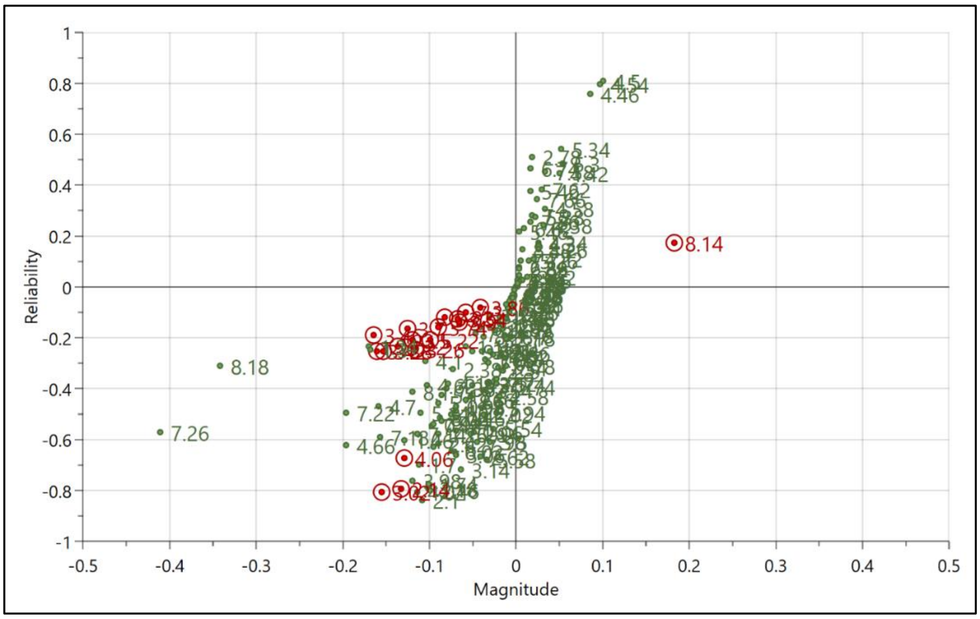

Figure S12. Corresponding S-plot with highlighted NMR spectral regions between control and metformin.

iv. **Control and Z_{lean} Diet A**

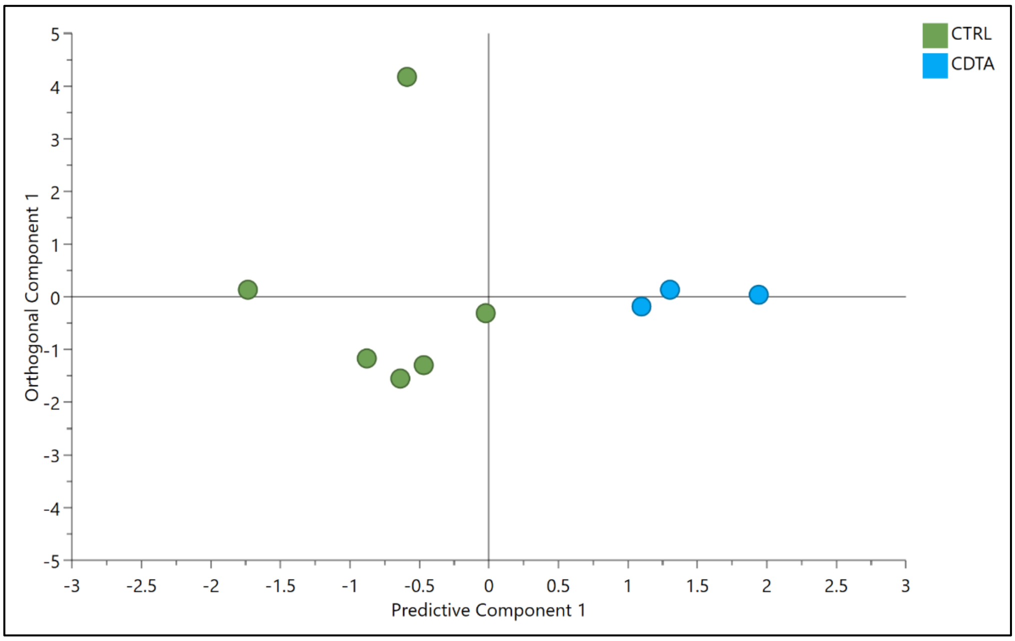

Figure S13. OPLS-DA score plot between ZDF control and Z_{lean} Diet A group.

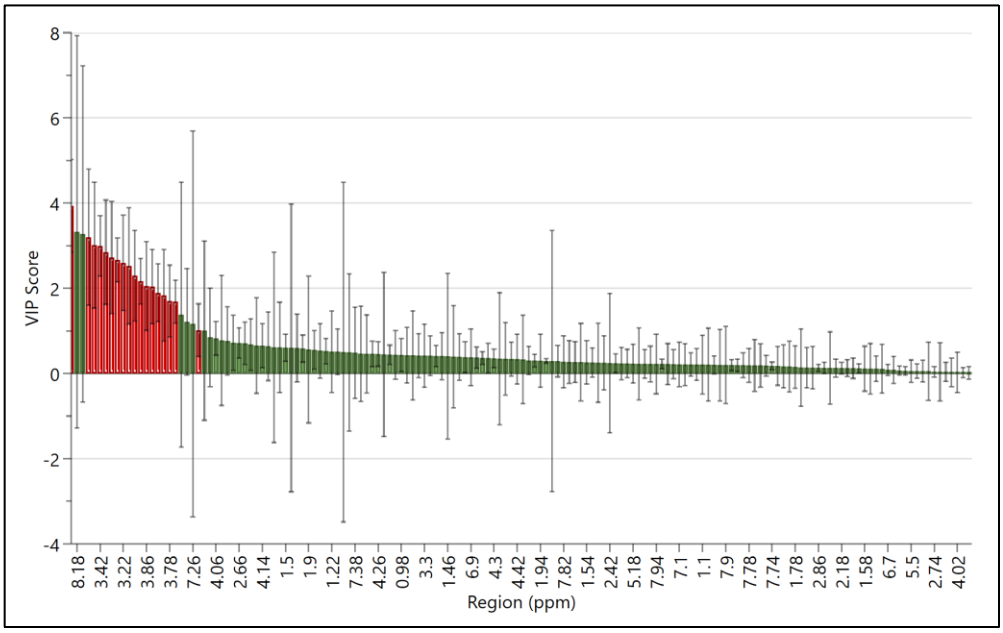

Figure S14. VIP plot for important NMR spectral regions (ppm) highlighted in red between ZDF control and Z_{lean} Diet A.

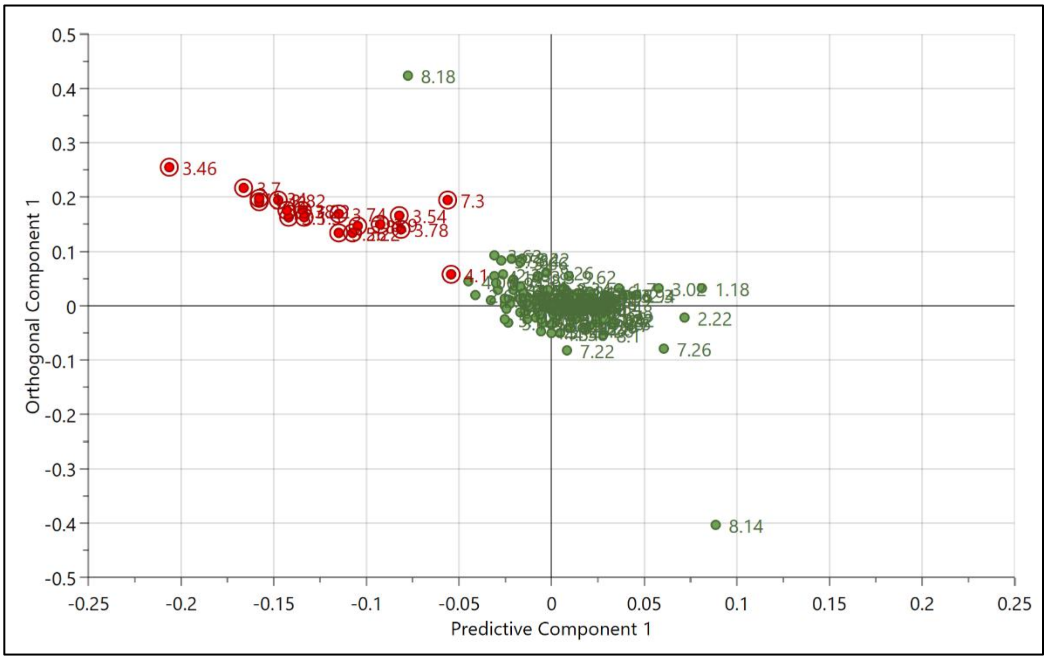

Figure S15. Corresponding loading plot with highlighted NMR spectral regions (ppm) highlighted in red between ZDF control and Z_{lean} Diet A.

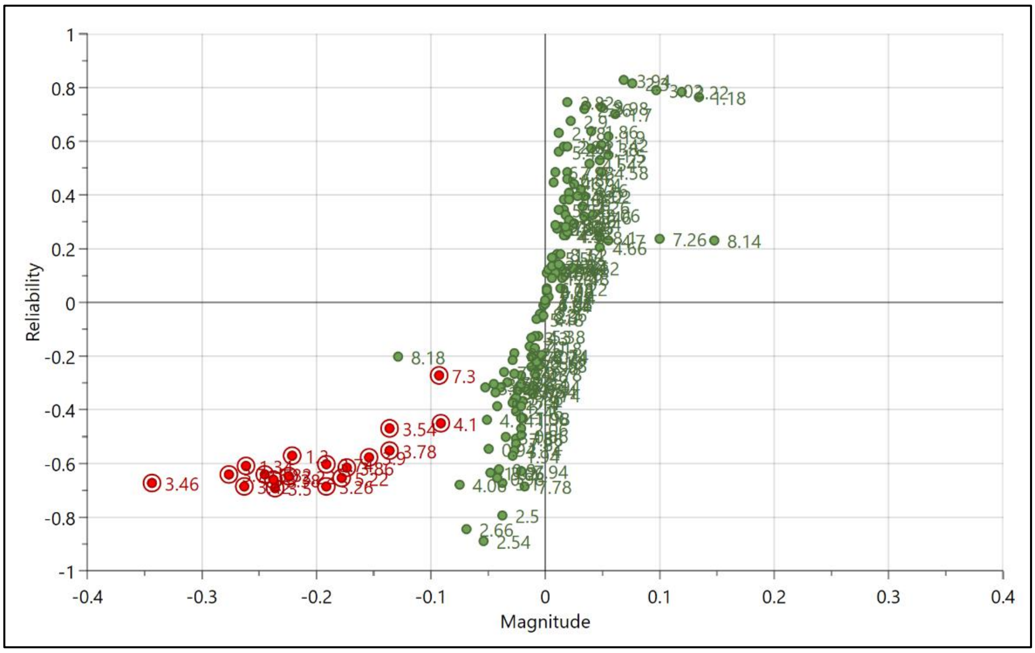

Figure S16. Corresponding S-plot with highlighted NMR spectral regions between ZDF control and Z_{lean} Diet A.

v. Control and Z_{lean} Diet B

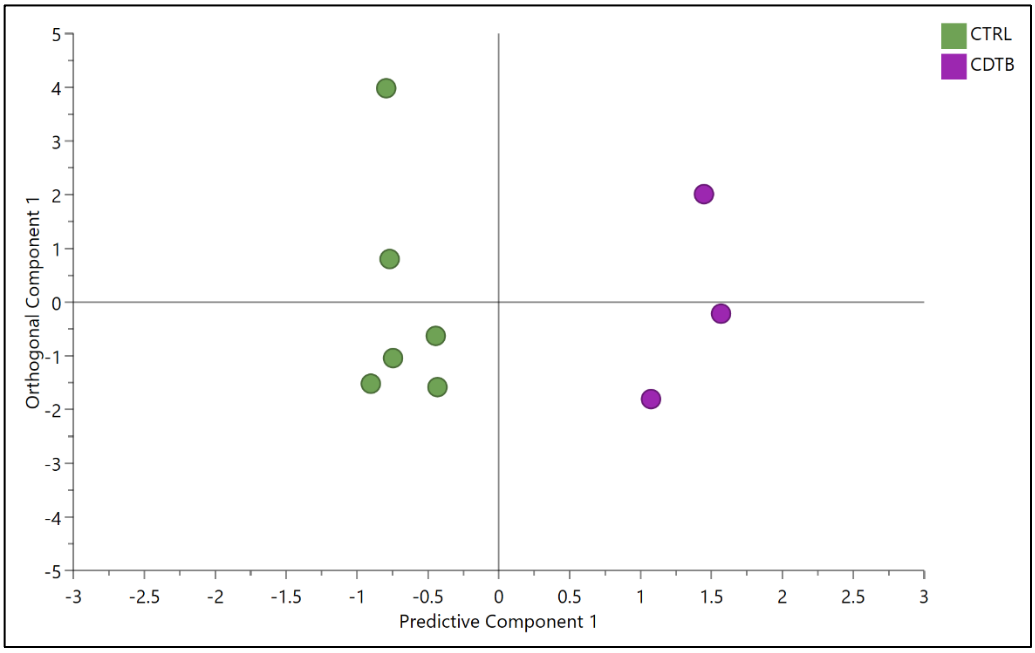

Figure S17. OPLS-DA score plot between ZDF control and Z_{lean} Diet B group.

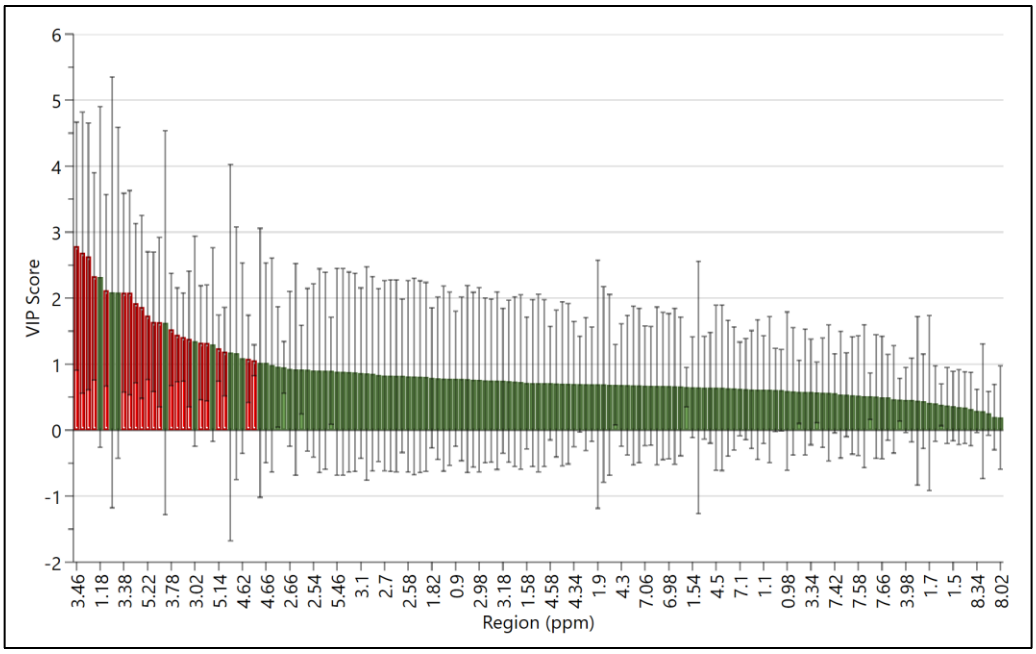

Figure S18. VIP plot for important NMR spectral regions (ppm) highlighted in red between ZDF control and Z_{lean} Diet B.

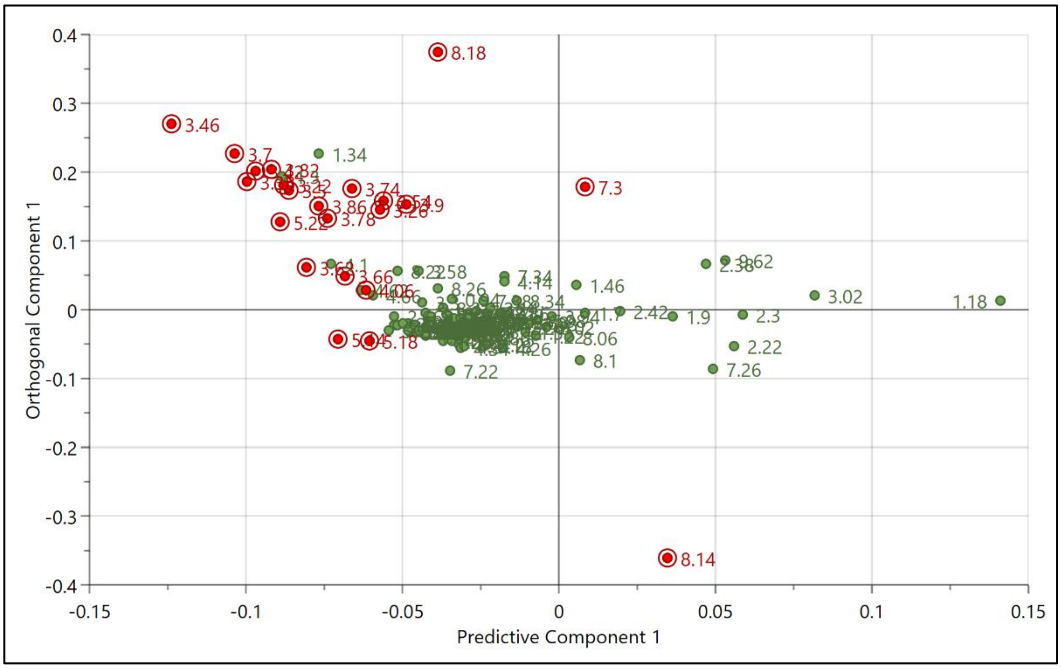

Figure S19. Corresponding loading plot with highlighted NMR spectral regions (ppm) highlighted in red between ZDF control and Z_{lean} Diet B.

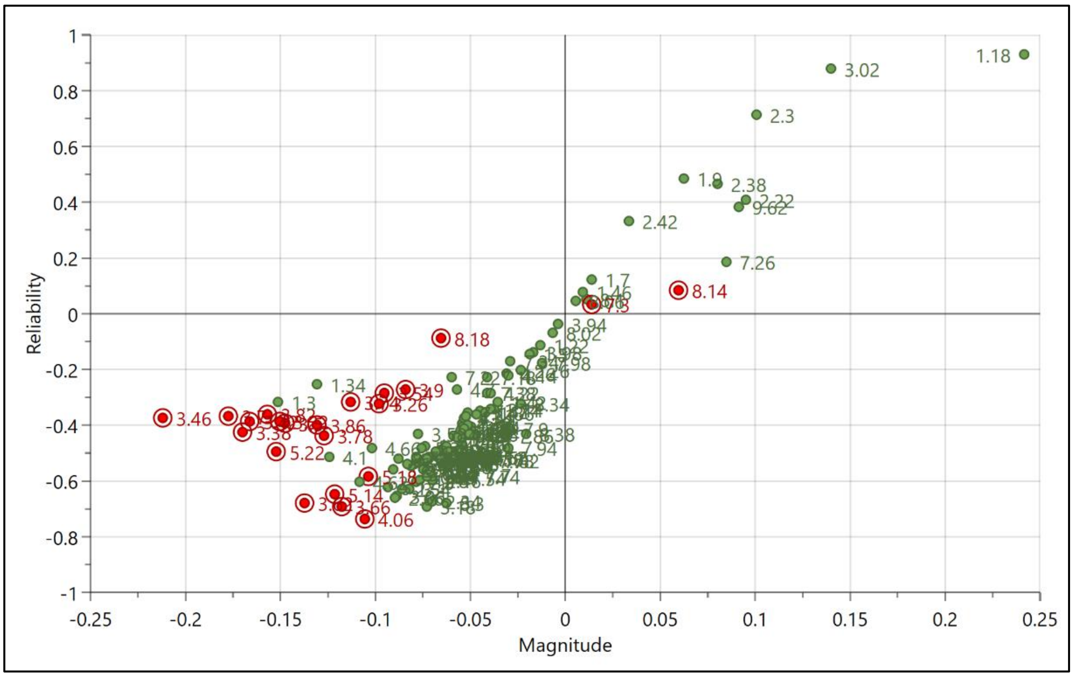

Figure S20. Corresponding S-plot with highlighted NMR spectral regions between ZDF control and Z_{lean} Diet B.

A supervised OPLS-DA analysis was performed for the ZDF and Z_{lean} treatment groups. The analysis revealed a clear separation between the metabolomic profiles of the treatment groups, although the predictive ability and statistical significance varied across comparisons. For the control vs. Diet A comparison, the model explained 72.2% of the variation but failed to provide valid predictions (Q^2=-0.282) and was not statistically significant (p>0.05). In contrast, the control vs. Diet B comparison accounted for 76.5% of the variation, with 95.4% due to treatment differences, and demonstrated good predictive power (Q^2=0.708), although it was not statistically significant (p>0.05). The control vs. metformin comparison showed a model explaining 73% of the variation, with 70.2% due to treatment differences, but failed to provide valid predictions (Q^2=-0.197) and was not statistically significant (p>0.05). For the control vs. Z_{lean} Diet A, the model explained 74.8% of the variation, with 82.3% due to treatment differences, and achieved a good fit, though predictions were not statistically significant (Q^2=0.08, p>0.05). Finally, the control vs. Z_{lean} Diet B comparison accounted for 90.6% of the variation, with 96.3% of this variation attributed to treatment differences, demonstrating good model fit (Q^2=0.666), though it was not statistically significant (p>0.05). Despite the lack of statistical significance in some cases, all analyses indicated distinct metabolomic separations between the groups.

Table S1. Distinguishing regions.

	ZDF Diet A	ZDF Diet B	ZDF Metformin	Z_{lean} Diet A	Z_{lean} Diet B
Distinguishing Regions (ppm)	1.18	1.18	3.46	3.22	3.38
	1.3	1.3	3.7	3.38	3.46
	2.22	1.34	3.82	3.42	3.66
	1.34	2.22	8.14	3.46	3.7
	7.3	2.3		3.7	4.06
		7.3		3.82	5.14

Table S2. Metabolites and spectra regions identified.

Metabolites	Spectra Regions (ppm)
1,6-Anhydro-β-D-glucose	3.5, 3.7, 4.1, 4.6, 5.4
2-Hydroxyvalerate	0.9, 1.3, 1.4, 1.6, 1.7, 4.0
2-Methylglutarate	1.1, 1.6, 1.7, 2.1, 2.2
2-Oxoglutarate	2.4, 3.0
3-Aminoisobutyrate	1.2, 2.6, 3.0, 3.1
3-Hydroxybutyrate	1.2, 2.3, 2.4, 4.1
Acetate	1.9
Agmatine	1.7, 3.0, 3.2, 7.2
Alanine	1.5, 3.8
Anserine	2.6, 2.7, 3.0, 3.2, 3.8, 4.5, 7.1, 8.2, 8.3
Cadaverine	1.5, 1.7, 3.0
Carnitine	2.4, 3.2, 3.4, 4.6
Choline	3.2, 3.5, 4.1
Citrate	2.5, 2.7
Creatine	3.0, 3.9
Formate	4.4
Gluconate	3.7, 3.8, 4.0, 4.1
Glucose	3.2, 3.4, 3.5, 3.7, 3.8, 3.9, 4.6, 5.2
Glutamate	2.0, 2.1, 2.3, 2.4, 3.7
Glutamine	2.1, 2.4, 2.5, 3.8, 6.9, 7.6
Glycine	3.6
Glyclyproline	1.8, 1.9, 2.0, 2.1, 2.2, 2.3, 3.5, 3.6, 3.9, 4.3
Histidine	3.1, 3.2, 4.0, 7.1, 7.9
Homocysteine	2.1, 2.2, 2.6, 2.7, 3.9
Hydroxyacetone	2.1, 4.4
Isoleucine	0.9, 1.0, 1.2, 1.5, 2.0, 3.7
Lactate	1.3, 4.1
Leucine	0.9, 1.0, 1.7, 3.7
Lysine	1.4, 1.5, 1.7, 1.9, 3.0, 3.7
Methionine	2.1, 2.2, 2.6, 3.9
N-Methylhydantoin	2.9, 4.1
Ornithine	1.7, 1.8, 1.9, 3.1, 3.8
Proline	2.0, 2.1, 2.3, 3.3, 3.4, 4.1
Putrescine	1.8, 3.0
Pyruvate	2.4
Succinate	2.4
Taurine	3.3, 3.4
Threonine	1.3, 3.6, 4.3
Trimethylamine N-oxide	3.3
Tyrosine	3.0, 3.2, 3.9, 6.9, 7.2
Valine	1.0, 2.3, 3.6
myo-Inositol	3.3, 3.5, 3.6, 4.1
trans-4-Hydroxy-L-proline	2.1, 2.4, 3.4, 3.5, 4.3, 4.7
π-Methylhistidine	3.2, 3.3, 3.7, 4.0, 7.1, 8.0
